# Nanogenerators in Biomedical Frontiers: Revolutionizing Self-Powered Healthcare Systems

**DOI:** 10.1021/acsomega.5c08225

**Published:** 2026-02-11

**Authors:** Anjali Varshney, Sunil Chauhan, Sangeeta Rawal, O. Raymond Herrera, Subhash Sharma

**Affiliations:** † Department of Physics & Environmental Sciences, Sharda School of Engineering & Science, 193167 Sharda University, Plot No. 32-34, Knowledge Park III, Greater Noida, Uttar Pradesh 201310, India; ‡ Centre for Solar Cell and Renewable Energy, Department of Physics & Environmental Sciences, Sharda School of Engineering & Science, Sharda University, Plot No. 32-34, Knowledge Park III, Greater Noida, Uttar Pradesh 201310, India; § Centro de Nanociencias y Nanotecnología, 87793 Universidad Nacional Autónoma de México, Km. 107 Carretera Tijuana-Ensenada, AP 14, Ensenada 22860, Baja California, Mexico; ∥ SECIHTI—IxM—Centro de Nanociencias y Nanotecnología, Universidad Nacional Autónoma de México, Km. 107 Carretera Tijuana-Ensenada, AP 14, Ensenada 22860, Baja California, Mexico

## Abstract

Self-powered systems have emerged as transformative technologies that address the growing demand for sustainable, autonomous, and miniaturized energy solutions for next-generation biomedical devices. Unlike conventional sensors and therapeutic platforms that rely on external power sources or batteries, self-powered nanogenerators—based on piezoelectric, triboelectric, and hybrid nanogenerators—can harvest biomechanical or environmental energy to enable continuous operation. This review highlights the basics of nanogenerator mechanisms and material innovations, extending to their strategic integration into advanced biomedical applications. Particular emphasis is placed on applications such as regenerative hair growth techniques using electrical stimulation, motion-triggered drug release patches that ensure precise and sustained delivery, biocompatible electronic skin (E-skin) for real-time physiological sensing, wearable devices for continuous health monitoring, sweat-resistant wearables, hearing aids, ligament strain and bladder sensors, respiration-driven monitors, smart eye sensors, and scaffolds for cardiovascular and bone tissue repair through bioelectric cues. By evaluating both the opportunities and challenges, including energy conversion efficiency, long-term biocompatibility, device stability, and large-scale fabrication, this review provides a balanced outlook on the future of self-powered biomedical systems. The insights presented herein not only underscore their clinical and technological relevance but also identify key research directions required to bridge the gap between laboratory prototypes and practical healthcare applications.

## Graphical abstract

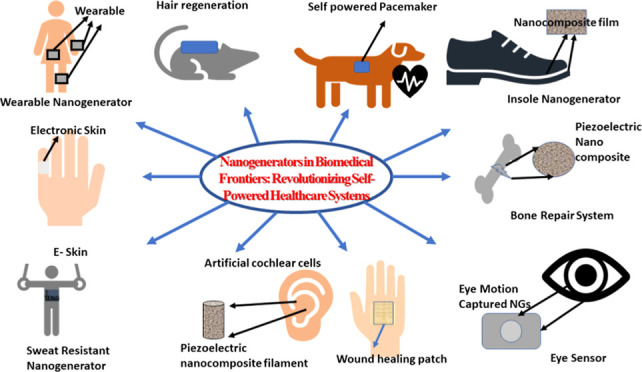

## 1 Introduction

With the growing urgency of the global energy crisis, the demand for clean and renewable alternatives to fossil fuels is increasing at an unprecedented rate. Historically, friction was one of the earliest known methods for generating electricity.^1,6^ In the past decade, rapid advancements have occurred in energy-harvesting technologies based on frictional (triboelectric) and piezoelectric principles. These mechanisms now serve as the foundation for nanogenerators (NGs) that are widely used to scavenge energy from natural sources such as wind, ocean waves, and mechanical vibrations.^2–5^ Simultaneously, the human body itself is a rich biomechanical energy reservoir, offering multiple avenues for sustainable energy harvesting. Nanogenerators have demonstrated the capability to harness energy not only from large-scale body motions to power wearable electronics but also from subtle physiological processes—such as heartbeat, respiration, and gastrointestinal movement—to power implantable medical devices (IMDs). These devices are designed to monitor or enhance the function of biological organs. Within this context, NGs act not only as energy sources but also as biomechanical sensors, converting organ motion into electrical signals for real-time diagnostic feedback. Conventional IMDs are typically powered by primary batteries, which present multiple limitations including frequent recharging or surgical replacement, finite lifespans, and environmental concerns related to toxic waste disposal. In contrast, human biomechanical energy offers a clean and continuous alternative. For instance, a person weighing 68 kg with 15% body fat contains approximately 384 MJ of bioenergy—derived from food metabolism—which is 35 to 100 times more energy-dense than conventional batteries. Even harvesting a small portion of this energy could sufficiently power low-energy devices used in wearable health monitoring.

Among mechanical energy harvesters, piezoelectric nanogenerators (PENGs) require repeated mechanical stress to produce electric charge, while triboelectric nanogenerators (TENGs) generate electricity through physical contact or friction. Compared to other energy-harvesting mechanisms, NGs offer key advantages such as material diversity, low cost, simple architecture, and high conversion efficiency, making them increasingly attractive in both scientific and biomedical fields.^7–11^

Since then, significant progress has been made toward miniaturizing nanogenerators while enhancing their peak power density and instantaneous conversion efficiency.^12^ These improvements enable efficient energy capture from periodic internal motions like pulmonary circulation, intestinal peristalsis, and skeletal muscle contractions.^13–18^ These physiological stresses, when paired with TENG/PENG operating principles, can convert mechanical deformations into digitally measurable signals, offering new tools for clinical diagnostics and patient monitoring.

This perspective provides a comprehensive overview of nanogenerator technologies for energy harvesting, beginning with a discussion of the fundamental operating principles of PENG and TENG systems. It then explores biomedical applications, such as their use in cardiovascular scaffolds, smart electronic skin for wound repair, and piezoelectric bone scaffolds. Finally, the review discusses the current challenges and future directions for the development of high-performance, clinically applicable nanogenerators, offering insights for ongoing and future research efforts.^19–25^ Furthermore, NGs like implantable piezoelectric and triboelectric nanogenerators (iPENG/iTENG) are essential in biomedical applications, including measuring ligament tension, powering implanted devices like pacemakers,^26^ cochlear implant,^27^ eye sensors,^28^ and targeted drug administration for diseases like cancer or tuber (Figure 1). They are perfect for next-generation self-powered systems because of their adaptability and versatility.

**Figure 1:**
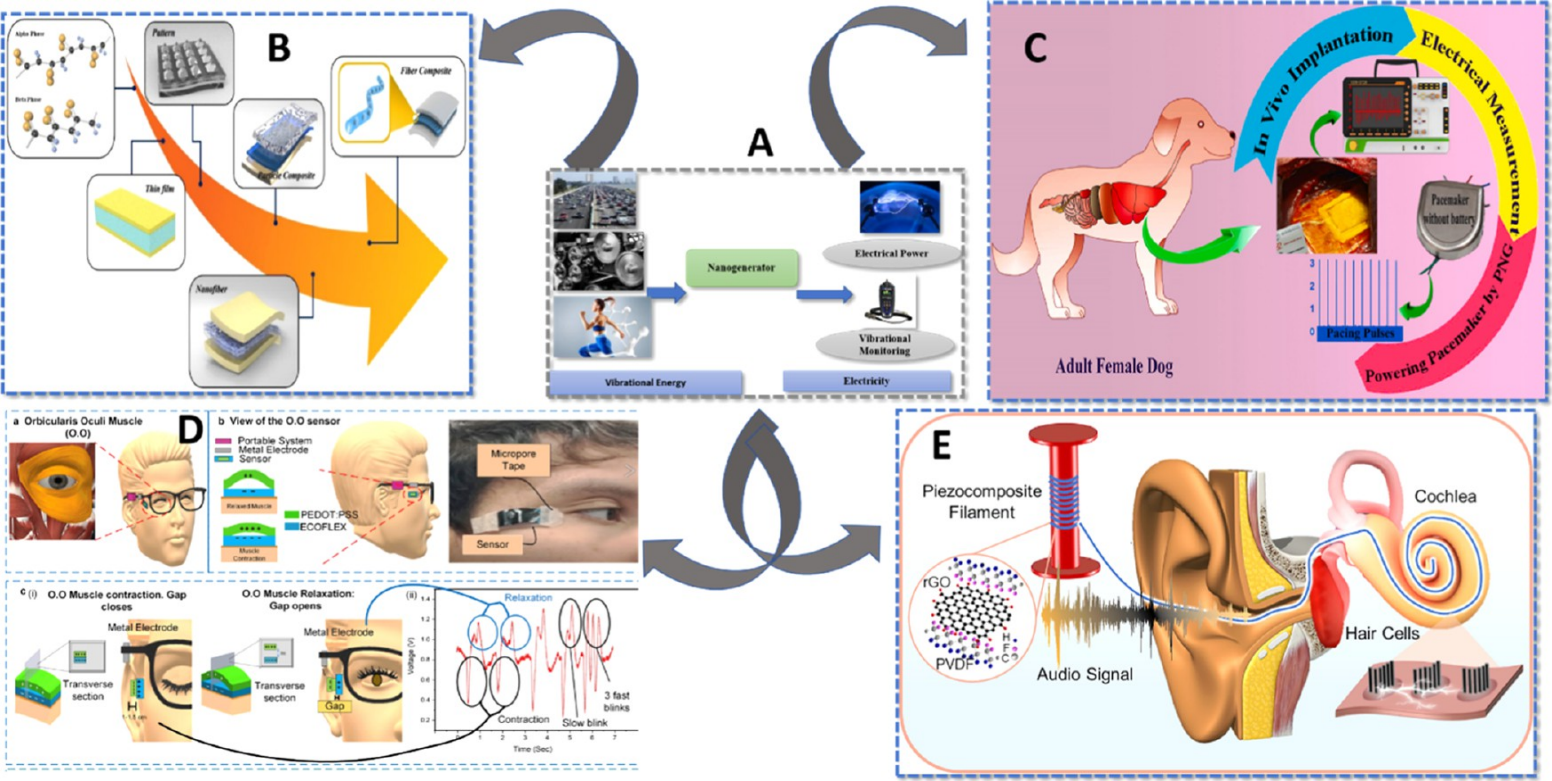
(A) Principle of nanogenerators. (B) Various structured polyvinylidene fluoride (PVDF)-based piezoelectric nanogenerators. Reproduced with permission from ref 26 Copyright 2020 MDPI. (C) Implant pacemaker in Adult female dog. (D) Design, positioning, and outcomes of eye motion sensors. (a) The area behind the eye is surrounded by the orbicularis oculi muscle. (b) Sensor positioning and overview. (c) (i) The muscle contracts and the sensor layers stretch when the eye is closed. Both the muscle and the sensor layers relax when the eye is opened again. It shows the transverse section. (ii) Signals of contraction and relaxation. Blinking slowly and quickly. Reproduced with permission from ref 28 Copyright 2020 Nano Energy. (E) Piezoelectric composite for cochlea implant. Reproduced with permission from ref 27. Copyright 2025 Energy & Environmental Materials.

The iPENG and iTENG generators make use of these small-sized devices, which are biocompatible and have the capacity to capture energy from vibrations or motions in the body for use in biosensing devices and medical implants. Although all varieties of nanogenerators use mechanical energy, the unique demands of the settings in which they function lead to substantial differences in their specific design concerns and uses. We thoroughly discuss the latest developments in polymer-based nanogenerators in this study, emphasizing their wide range of uses and documented performance results. Particular attention is paid to the incorporation of lightweight and flexible polymers in the creation of nanogenerators, which provide notable benefits in terms of mechanical robustness, biocompatibility, and flexibility. Here, the complete discussion of their newfound uses in the biomedical industry includes their use in therapeutic applications like targeted medication administration and in powering tiny implanted devices like pacemakers and biosensors. Additionally, the paper discusses wearable technology that transforms bodily movements into electrical energy that can be utilized, like respiration monitoring systems and insole-based energy harvesters. These advancements demonstrate the enormous promise of polymer-based nanogenerators in the creation of effective, self-sufficient systems for wearable electronics, healthcare, and other fields.

## 2 Origin of the Nanogenerator

In 1880, there are some theoretical insights explained by brothers Pierre and Jacques Curie, who also explained some theoretical insights. Both brothers discovered the piezoelectric effect. It involves physically transferring mechanical stress to electrical energy, which is limited to materials based on the dispersion of ions. The electric dipoles present in materials with a nonsymmetric ion distribution produce piezoelectric signals. When no external forces operate on a crystal structure, it is in a state of equilibrium between positive and negative electric charges or neutrality. Stress instantly alters the charge of the cations and anions in the center, leading to a change in polarization.

In another way, understanding the basic theoretical underpinnings of nanogenerators, particularly those based on piezoelectric, triboelectric, and electromagnetic principles, is made possible by Maxwell’s equations. By controlling the electric and magnetic fields in materials, these equations explain how mechanical energy is transformed into electrical energy in the settings of nanogenerators. For example, mechanical stress causes the material in piezoelectric nanogenerators (PENGs) to polarize, producing time-varying electric displacement field D, Figure 2a. The schematic representation of the origin and evolution of the nanogenerator concept comes from Maxwell’s displacement current theory. The left side (blue) represents the classical displacement current induced by the variation of the electric field (as described by Maxwell in 1861–62), which explains electromagnetic wave propagation and forms the root of modern wireless communication and photonic technologies. The right side (orange) illustrates the extension of Maxwell’s framework by Wang,^29^ introducing the polarization-driven displacement current, where strain-induced polarization variation generates current. This principle underpins the invention of piezoelectric and triboelectric nanogenerators (post-2006) and their wide-ranging applications in self-powered sensors, internet of things (IoT), robotics, and clean energy technologies. In other words, the fundamental origin of nanogenerators can be directly traced to the concept of displacement current in Maxwell’s equations. In classical electromagnetism, the term

$$\frac{\partial D}{\partial t}$$

represents the displacement current that explains the existence of electromagnetic waves, where *D* is the electric displacement field. However, Wang extended this concept by introducing the additional polarization-related term

$$\frac{\partial P_{s}}{\partial t}$$

which arises from the time-varying polarization in dielectric or piezoelectric materials under mechanical deformation. This new interpretation not only complements Maxwell’s original framework but also provides the theoretical basis for the working mechanism of nanogenerators.

**Figure 2:**
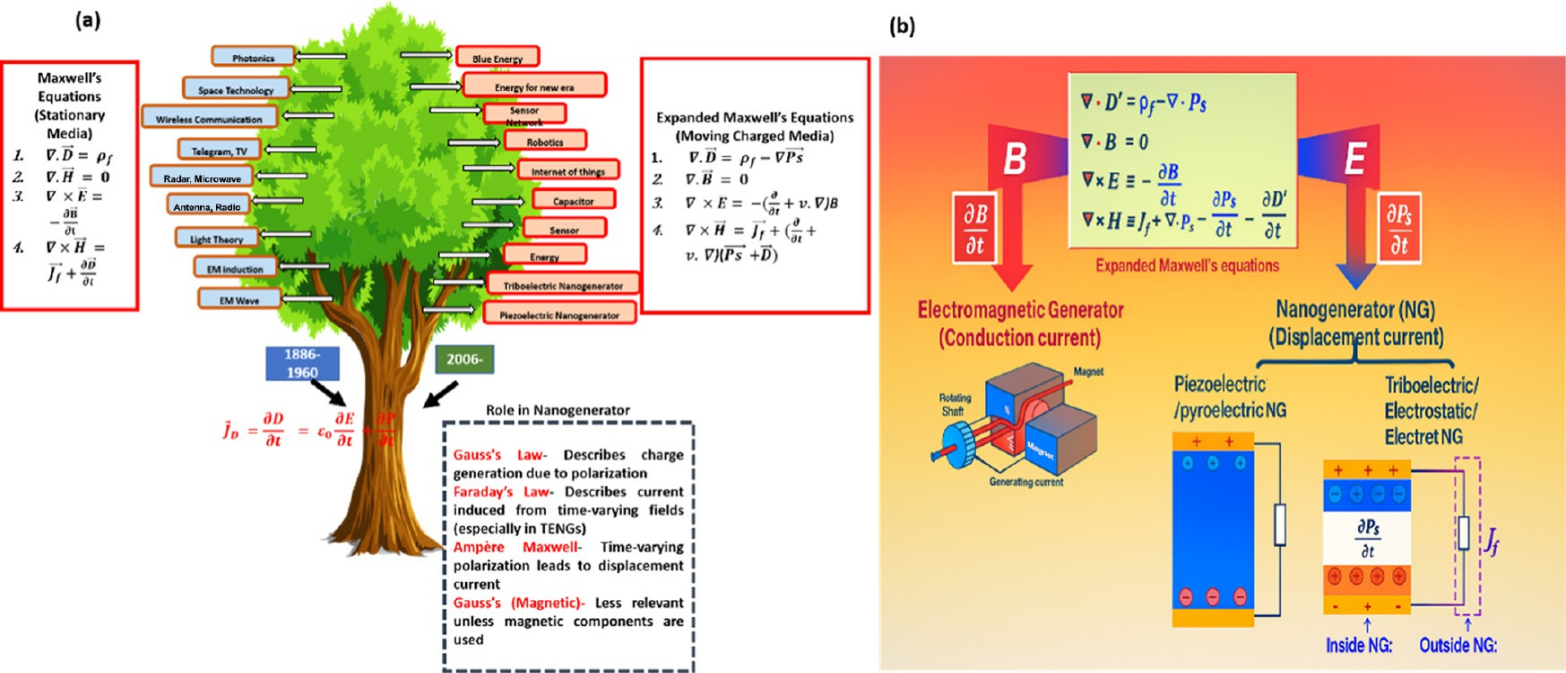
(a) Basic theory of nanogenerators. Reproduced with permission from ref 29 Copyright 2022 Materials Today. (b) A comparison of traditional electromagnetic generators and triboelectric, piezoelectric, and hybrid nanogenerators regarding the governing physics laws, types of currents, and their representing physical quantities in the expanded Maxwell’s equations. Reproduced with permission from ref 29. Copyright 2022 Materials Today.

As illustrated in Figure 2b, conventional electromagnetic generators (EGs) rely on the conduction current (*J*_c_) generated through the relative motion of a coil and magnetic field, typically requiring large-scale mechanical input. In contrast, nanogenerators (NGs) operate on the principle of displacement current generated at the nanoscale, where strain-induced polarization (piezoelectric effect) or charge transfer through surface contact electrification (triboelectric effect) drives current flow. In piezoelectric nanogenerators, the dynamic strain alters the spontaneous polarization within the crystal lattice, producing an alternating potential difference. In triboelectric nanogenerators, surface charge exchange during contact and separation produces a varying electric field, which equivalently acts as a displacement current.

This distinction is critical because it highlights why NGs are fundamentally different from EGs: while EGs harvest energy from macroscopic electromagnetic induction, NGs harvest energy from nanoscale polarization dynamics. The expanded Maxwell’s equations unify both concepts, showing that NGs are a natural extension of electromagnetic theory into the nanoscale regime. Importantly, this interpretation has laid the foundation for a new field of self-powered nanosystems, enabling diverse applications in biomedical devices, human-machine interfaces, soft robotics, and IoT. Also, Gauss’s law for electricity states that this displacement causes free charges to form on the electrodes, which results in current. Maxwell’s displacement current theory is especially significant in triboelectric nanogenerators (TENGs). It describes how current flows in the external circuit as a result of time-varying surface charge distributions brought on by contact electrification and the relative motion between two materials. Furthermore, Faraday’s equation of induction is essential in electromagnetic nanogenerators (EMGs), where an electromotive force (EMF) is induced by shifting magnetic fields brought on by motion. Together, these equations describe how nanogenerators couple mechanical motion with field fluctuations in space and time to transform various mechanical inputs into useful electric power.^29^

A nanogenerator is a device that can transform mechanical or thermal energy into electrical energy on the nanoscale. It usually consists of nanoparticles with the ability to produce electricity via pyroelectricity (property of some materials to produce an electric charge when their temperature varies), triboelectricity, or piezoelectricity, among other methods. With the capacity to gather energy from ambient sources, including temperature changes, vibrations, and body motions, nanogenerators show great promise for use in wearable electronics, wireless sensors, and self-powered electronics. By offering nanoscale renewable energy solutions, these nanoscale power sources aid in the development of autonomous and sustainable systems. Based on the method of electrical power harvesting, Figure 3 primarily divides them into two groups: piezoelectric nanogenerators (PENG) and triboelectric nanogenerators (TENG). The concepts relate to the corresponding TENG and PENG phenomena. HNGs, or hybrid nanogenerators, mostly relate to nanogenerators using these two in concert and/or other consequences like thermal electricity.^30^

**Figure 3:**
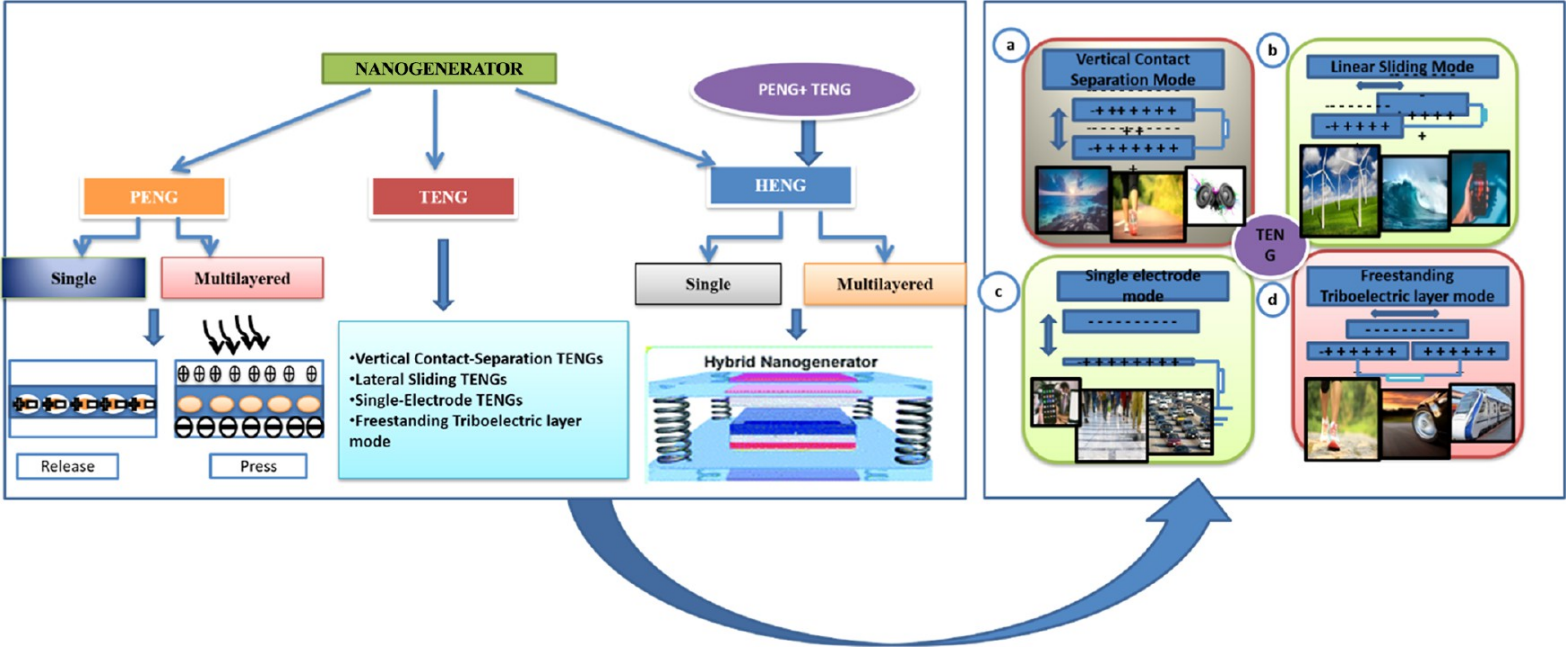
Types of nanogenerators like PENG, TENG, and HNG with the different modes of TENG.

### 2.1 Piezoelectric Nanogenerator (PENG)

PENG is a device that uses the effect of piezoelectrics for the transformation of mechanical energy to electrical energy (Figure 3). The phenomenon of some materials producing an electric charge in response to an applied mechanical stress is defined as the piezoelectric effect. A nanogenerator utilizes this effect at the nanoscale to generate small yet useful electrical power output. The potential difference appearing between the two electrodes through self-polarization controls the stream of electrons between electrodes through an external load. Maxwell’s equations may be used to demonstrate how NG devices operate. Maxwell’s equations in electrodynamics are constant when there is local invariance, which may be written as:

i $$\nabla .\mathrm{J⃗}+\frac{\partial \left(\nabla .\mathrm{D⃗}\right)}{\partial t}=0$$

ii $$\frac{\partial \rho }{\partial t}+\nabla .\mathrm{J⃗}=0$$

Given equation is known as the equation of continuity, where total charge density (ρ) and total current density (*J*) are the respective values. One way to define the charge density is

iii $$\rho =\rho_{0}+\rho_{b}$$

where, ρ_b_ represents bound charges and ρ_0_ represents free charges.

The current density can be stated as

iv $$J=J_{0}+J_{b}$$

where, *J*_b_ represents bound currents and *J*_0_ represents free currents. When it comes to dielectric materials, the auxiliary fields actively participate in the creation of dipoles and are characterized as

v $$D\left(r,t\right)=\varepsilon_{0}E\left(r,t\right)+P\left(r,t\right)$$

$$H\left(r,t\right)=\frac{B\left(r,t\right)}{\mu_{0}}-M\left(r,t\right)$$ vi

In this case, *D* notifies for displacement field, *E* for electric field, ε_0_ for free space permittivity, *H* for magnetic field, *P* for polarization, *B* for magnetic induction, μ_0_ for free space permeability, and *M* for magnetization. The fundamental idea behind our current study may be understood by using Maxwell’s equations for matter. Taking into account the current and free charges, Maxwell’s equations given below

vii $$\nabla .\mathrm{D⃗}=\rho_{f}\left(Gauss’s\;Law\;of\;electro\;statics\right)$$

viii ∇.H⃗=0(Gauss’slawmagnetostatics)

ix $$\nabla \times \mathrm{E̅}=-\frac{\partial \mathrm{B⃗}}{\partial t}\left(Faraday’s\;law\right)$$

x $$\nabla \times \mathrm{H⃗}=\vec{J_{f}}+\frac{\partial \mathrm{D⃗}}{\partial t}\left(Ampere’s\;circuital\;law\;with\;correction\right)$$

In this case, *J*_f_ represents the Free current density caused by the free electron flow, ρ_f_ represents the volume charge density of the free electron, *E* represents the electric field, *D* represents the displacement electric field, *H* represents the magnetic field, and *B* represents magnetic induction. The following formulas can be used to derive the displacement current corresponding to an electric field and also the polarization current corresponding to an electric polarization from electrodynamics. The polarization current for an electric polarization and displacement current for an electric field may be attained from electrodynamics using the following formulas.

xi $$J=\varepsilon_{0}\frac{\partial E}{\partial t}$$

xii $$J_{p}=\frac{\partial P}{\partial t}$$

Therefore, in the case of a linear isotropic media, the total displacement current ( ${\mathrm{J⃗}}_{D}$ ) is displayed here by the second term of Maxwell’s fourth equation

xiii $${\mathrm{J⃗}}_{D}=\frac{\partial D}{\partial t}=\varepsilon_{0}\frac{\partial E}{\partial t}+\frac{\partial P}{\partial t}$$

Thus, the dielectric medium’s electric polarization and electric field both affect the total displacement current, which is a time-dependent quantity. The piezoelectric equations for a linear isotropic material under mechanical strain are:^31^

xiv $$\vec{P_{i}}={\left(e\right)}_{ijk}{\left(\mathrm{S⃗}\right)}_{jk}$$

xv $$\mathrm{T⃗}=C_{E}\mathrm{S⃗}-e^{T}\mathrm{E⃗}$$

xvi D⃗=eS⃗−kE⃗

where, *S⃗* is mechanical strain, (e)_*ijk*_ is piezoelectric third-order tensor, *k* is dielectric tensor, *T⃗* is stress tensor, and *C*_E_ is elasticity tensor. In a linear polarizing media, the displacement current is

xvii $${\mathrm{J⃗}}_{Di}=\frac{\partial \mathrm{P⃗}}{\partial t}$$

Thus, output current density and piezoelectric effect are exactly dependent on the rate of change of strain or applied force, according to equation xvii.^26^ When the dielectric material is not subject to an external field E, the current of displacement is solely dependent on polarization, as stated in equation xviii. Here, considering the polarization along the *z*, this may be written as

xviii $$D_{z}=P_{z}=\sigma_{P}\left(z\right)$$

where, σ_P_(*z*) is piezoelectric polarization and surface charge density. Then, the displaced current term along *z* direction will be

xix $$J_{DZ}=\frac{\partial P_{Z}}{\partial t}=\frac{\partial \sigma_{p}\left(z\right)}{\partial t}$$

The equation above explicates the rationale behind the outstanding performance of the NG gadget.^32^

When pressure is applied to a material, it creates both positive and negative charges on its crystal surface, which are called piezoelectric materials. Pyroelectric crystals exhibit spontaneous polarization and uneven positive and negative charges in their crystal structure when no applied electric field acts on the material. But when an electric field reverses the spontaneous polarization of dielectrics, as long as no stronger electric field is introduced that may rupture the crystal limit, these materials are called ferroelectrics.^33–35^ First, consider piezoelectric-based NGs, which have two types of piezoelectric effects: the direct effect and the inverse effect. The direct piezoelectric effect can only be explained by Hooke’s Law, which establishes a basic relationship between the stress placed on a material and the strain it experiences as a consequence. Understanding the significance of this linear connection is crucial to understanding how mechanical stress generates an electric charge in piezoelectric materials. Materials adhering to Hooke’s law exhibit predictable deformation behavior in response to applied stress, enabling precise control and measurement of the piezoelectric effect. Hook’s law gives the simplified equation as

xx $$S_{i}=s_{ij}^{E}T_{j}+d_{im}E_{m}$$

whereas, *S* represents the strain and *s* ^E^ represents the mechanical compliance at a constant electric field. The piezoelectric constant is *d*, the stress is *T*, and the electric field is *E*. The process is different when it comes to the inverse piezoelectric effect: initially, an electric potential is supplied, which results in certain mechanical alterations in the material structure. According to that the equation suggests:

xxi $$D_{k}=d_{jk}T_{j}+e_{km}^{T}E_{m}$$

where *m* denotes rotational motion along the three axes, *D* stands for the electric displacement, and *i*, *j*, and *k* are distinct directions in the material (coordinates system). The observed complicated mix of electrostriction, piezo-striction, and strain due to domain reorientation with hysteresis may explain the origin of induced strain. In piezoelectronics, the performance of actuators, transducers, and other devices is often evaluated using a figure of merit that considers five key elements. These elements are: (1) mechanical quality factor *Q*_m_; (2) electromechanical coupling factor (k); the coefficient of energy transmissions (λ); and the efficiency (η); (3) acoustic impedance (*Z*); (4) piezoelectric coefficient *d*, and *g*; and (5) maximum vibration velocity ν_max_.^29,30^ The volume change that happens when a material is exposed to an electric field or the polarization that it experiences when mechanical stress is applied is referred to as the piezoelectric coefficient (d), which may be used to determine the piezoelectricity of various materials. The equation is as follows:

xxii $$d=\frac{P}{\sigma }$$

stated by the unit C/N due to p and s are the polarization and stress in units of C/m^2^ and N/m^2^],^36^ respectively. The following additional constants help us understand how piezoelectric materials behave: (1) piezoelectric voltage constant (g): electric field produced when a unit force is applied to a piezoelectric material. It is expressed in [Vm/N] units and is mostly used to evaluate the material’s macroscopically sound performance.

(2) The piezoelectric performance is changed when the shape of a material changes. The number of charges for a particular strain (*e*_ij_), which is expressed in [C/m^2^] units, is the cause of this.^37^ Ohmic and Schottky connections enable the harvesting of piezoelectric energy from the electrode material. The superposition of all of the dipoles inside the crystal that results in a significant potential differential along the polarization direction is called a piezopotential. At the opposite junction, which features an ohmic contact, current passes through the piezoelectric material in a DC nanogenerator in one direction. A piezopotential is generated at the Schottky contact that is larger than the inherent Schottky barrier height. However, an AC nanogenerator results from a Schottky barrier height that is too high to overcome, which causes the Schottky contact to close and cut off the current flow of the external circuit. The current returns upon the release of the imposed tensions. Therefore, the in situ rectifying behavior of the Schottky contact determines the output form of PENGs.^38^ Surface desorption and inherent flaws in the piezoelectric materials generate free-charge carriers. In addition to Coulombic interactions, the polarization field can also cause free carrier redistribution and affect the energy band structures at the interface. Consequently, external mechanical forces direct the charge transport mechanism near the interface. As a result, there is a noticeable discontinuity in energy levels where piezoelectric semiconductors and other reactants meet.^39^ Fermi levels between the two electrodes in nanogenerators must line up. To achieve an electrostatic level that is balanced, the charge carriers must flow in the direction of an outside load. This is because stresses cause a piezoelectric potential between the inside and outside Fermi levels, which makes a potential difference.^36,40^ While the piezoelectric device is operating due to the configuration, there is only one piezoelectric strain coefficient that dominates the output. Two primary modes of operation, 33 and 31, determine the application of stress in the field of piezoelectric energy harvesting based on the direction of polarization in the piezoelectric material. Mode 33 delivers stress parallel to polarization, causing direct charge separation between the electrodes. Instead, shear deformation and charge displacement are the outcomes of the mode 31 application of stress perpendicular to polarization. Based on mechanical stimuli and material qualities, understanding these modes aids in optimizing energy conversion efficiency.

The following equation gives the voltages *V* and *E* resulting from applied stress:

xxiii $$V=\frac{d_{33}}{e_{33}^{T}}h\Delta \sigma$$

xxiv $$E=\frac{1}{2}C\frac{d_{33}^{2}}{e_{33}^{T}}At\Delta \sigma^{2}$$

These equations demonstrated the relationship between the thickness of material with its capacitance *C*, its area of piezoelectric material, and its permittivity under constant stress in 33-mode. Piezoelectric materials having a high-harvesting figure of merit $\frac{d_{33}^{2}}{e_{33}^{T}}$ can be utilized to optimize the amount of energy collected, depending on the thickness and area. Piezoelectric charge constant (*d*_33_) values are often greater for 33 arrangements. Materials with strong electromechanical coupling coefficients and those that can be readily deformed to produce greater stresses are favorable for energy harvesting.^34^

The following formula xxv, can be used to get the electromechanical coupling factor (*k*), which states the energy conversion efficiency, for a certain material: Young’s modulus (*Y*), elastic energy stored in the material (*W*), electrical energy produced by stress on the piezoelectric element (*E*_c_), dielectric constants (ε), and piezoelectric constants (*d*) are all shown.^41^

xxv $$k^{2}=\frac{E_{c}}{W}=Y\frac{d_{SS}^{2}}{e}$$

Also expressed as in^33^, converted energy is equivalent to *k* ^2^.

Properties of piezoelectric materials can be used to define their range of uses. Poly­(vinylidene fluoride) (PVDF) composite nanofibers doped with a hybrid nanofiller of reduced graphene oxide (rGO) and zinc oxide (ZnO) illustrate how piezoelectric nanogenerators can be purpose-built for biomedical use. Electrospinning PVDF into flexible fibrous mats already promotes the electroactive β-phase essential for piezoelectricity, but infusing the fibers with rGO sheets furnishes conductive pathways that lower internal impedance and speed charge collection, while embedded ZnO nanoparticles introduce additional noncentrosymmetric domains that couple mechanical strain directly into polarization. The synergistic filler combination therefore boosts the output voltage and current far beyond neat PVDF, allowing the nanogenerator to scavenge biomechanical energies as small as chest-wall expansion during breathing or pulsatile blood-vessel motion. In wearable form, a thin patch laminated to the sternum can continuously power low-power wireless respiratory or ECG sensors; implanted against bone or fascia, the same material harvests micromechanical stresses to drive localized electrical stimulation that accelerates osteogenesis or wound repair. Because both rGO and ZnO are chemically stable and, in low loading, biocompatible, they preserve cytocompatibility while reinforcing the fiber network’s tensile strength and fatigue life—two critical attributes for long-term operation in the dynamic, aqueous environment of the human body.^41^

### 2.2 Triboelectric Nanogenerator (TENG)

A device created as a triboelectric nanogenerator (TENG) using the triboelectric effect and electrostatic induction converts mechanical energy into electrical energy. When several materials come into contact with one another, separate, and then become electrically charged, this phenomenon is known as the triboelectric effect. A TENG produces an electric current by bringing two materials with dissimilar properties into contact and then separating them. This creates a potential difference by applying mechanical force, such as pushing, tapping, or rubbing. TENGs are used in biomedical devices, wearable electronics, self-powered systems, and energy-harvesting technologies. The configurations are illustrated in Figure 3. For instance, the application of marine biomaterials extends beyond serving as a triboelectric layer in TENGs. These biomaterials play a crucial role in creating transparent and stretchable TENGs utilizing hydrogel electrodes. Such TENGs show great promise in areas such as energy collection, environmental management, and biomedical implantable devices. Li et al. 2023 explained the operational mode of TENG, which is based on four fundamental modes (Figure 3), differentiated by the way the triboelectric layers interact to promote the electrical induction operation and by the layout of the electrodes.^42^ Various modes of TENG are described in detail.

(i) Contact-Separation (CS) Mode

The CS mode uses two triboelectric and two electrode layers in its basic arrangement. The cyclic connection and detachment of these layers drive an outside current to balance the potential distinction between the electrodes, causing the potential difference between the electrodes to periodically emerge and dissipate.

(ii) Linear-Sliding (LS) Mode

In contrast to the CS mode, the LS mode produces triboelectric charges by lateral motion at the contact region interface. The primary purpose of these TENGs, particularly those having a radial grating disk structure, is to collect the flow and rotational energy. Because of the high-frequency, continual relative sliding that compromises TENG integrity, there are still concerns about the long-term durability of these systems, despite their notable power output.

(iii) Single-Electrode (SE) Mode

In order to collect energy from free objects that move without the necessity for electrode obsession, the SE mode makes use of a single electrode. Though this mode is perfect for developing autonomous touch sensors or human-machine interfaces, its relatively modest electrical output can be attributed to restrictions resulting from the main electrode’s electrical shielding effect.

(iv) Freestanding Triboelectric-Layer (FT) Mode

A symmetrical pair of electrodes and a movable triboelectric layer are involved in the FT mode. There is periodic movement of the layer between electrodes due to electron movement. An alternating current (AC) output is produced. The FT mode, which is highly regarded for its exceptional energy transformation efficiency, finds use in several domains, including rotational energy harvesting, wave energy, and vibrational energy. Though each mode has advantages of its own, it is important to remember that TENGs can use more than one mode in real-world situations. Combining several modes offers a way to take advantage of their combined advantages, guaranteeing maximum performance in a range of applications.

### 2.3 Hybrid Nanogenerator (HNG)

A hybrid nanogenerator can use both piezo and tribo nanomaterials to yield mechanical energy from motion and vibration. Triboelectric materials produce electricity by separating and contacting different materials, whereas piezoelectric materials transform vibration into electrical energy. A hybrid nanogenerator may more effectively capture mechanical energy and produce a more consistent power output by combining these two methods. In distant or unreachable areas where traditional power sources are unfeasible, hybrid nanogenerators have great potential for powering wearables, biomedical implants, sensors, and small-scale electronic equipment. They are useful instruments for developing energy-collecting technologies and meeting the increasing need for self-powered devices for a range of applications because they are versatile and adaptable. This makes them multidirectional energy-harvesting devices. Combining at least two energy-gathering methods is known as hybridization. All things considered, this pushes NG performance and efficiency to their absolute maximum.^43–45^ Triboelectric and piezoelectric components combined with the piezoelectric effect are a means of creating sophisticated hybrid nanogenerators.

## 3 Fabrication Technique for Polymers and Its Composite-Based Nanogenerator Electrodes

Polymers and their composites are fabricated by using a variety of procedures designed to produce materials with certain structures and qualities. Solution casting, electrospinning, and additive manufacturing are examples of common methods for 3D printing. The composition, morphology, and structure of the polymer and composite materials may be precisely controlled by using these techniques. Furthermore, the methods of surface modification and functionalization are frequently utilized to customize the characteristics of materials for particular uses. All things considered, these manufacturing methods are essential to the development of classy materials for various application purposes such as energy-harvesting systems, electronics, and medical equipment. Polymer composite-based nanogenerators have been realized using a variety of advanced processes, with examples of solution casting, electrospinning, and spin coating being noteworthy approaches. All the methods are illustrated in Figure 4. These methods have proven to be efficient for creating nanogenerators, allowing polymer composite materials to be integrated to produce useful devices with improved power-generating capabilities.^46–61^

**Figure 4:**
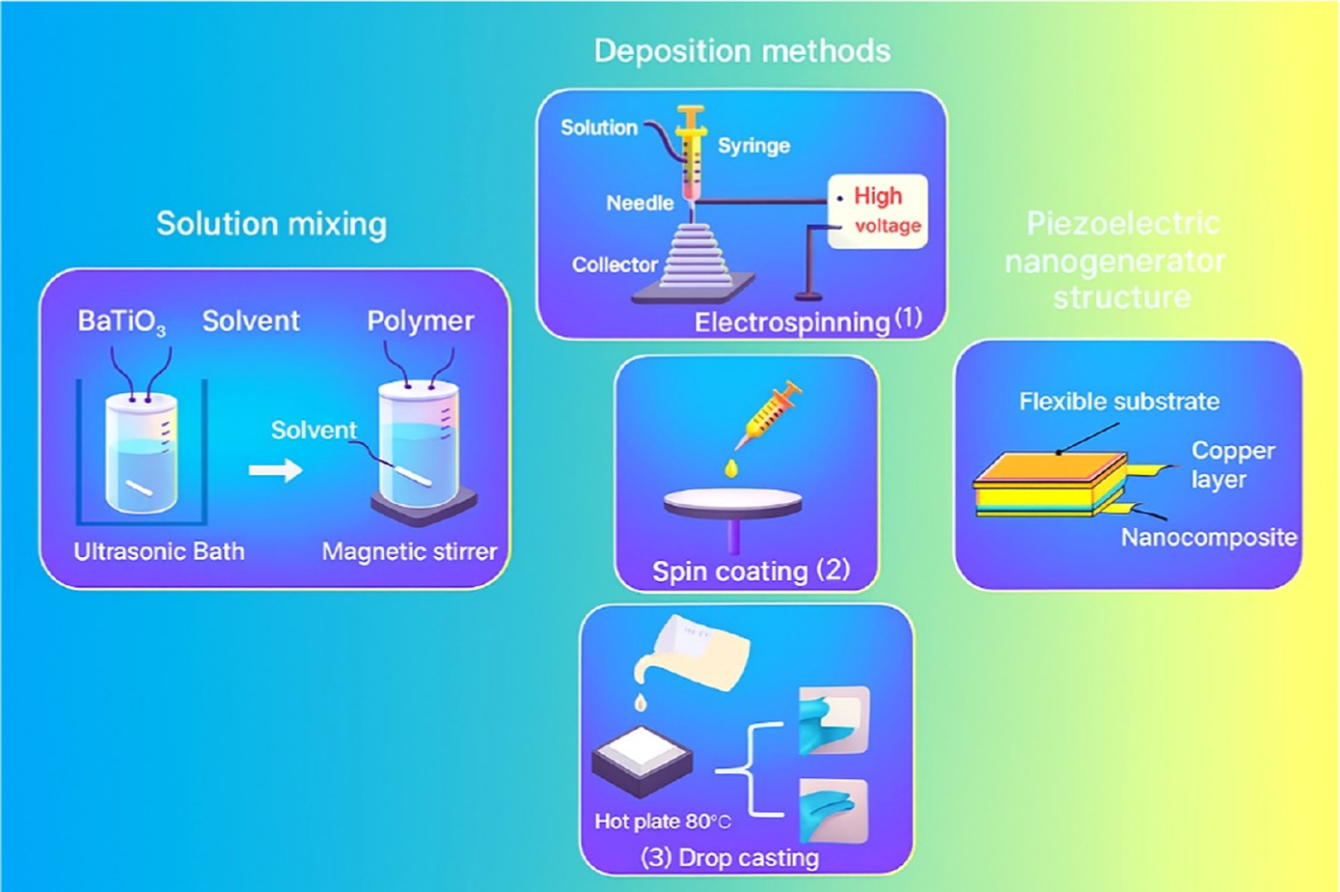
Schematic fabrication processes of nanogenerators based on BaTiO_3_ polymer composites. Reproduced with permission from ref 46. Copyright 2024 MDPI publishing.

## 4 Key Functional Materials for Nanogenerators

Many scientists have found that the initiation of various types of doping of physical conductive and nonconductive filler mixing quantities plays a major part in enhancing the piezoelectric result of PVDF-based NG. Also, inorganic filler polymers with high dielectric loss and conductivity become the reason for reducing discharge efficiency and conductive loss in nanocomposites; hence, only ceramics are needed, which have a moderate dielectric loss.^62^ Pure PVDF has limitations and exhibits a low dielectric constant. If a high-permittivity doping element is used with PVDF, it shows issues with dielectric compatibility, which means that interfacial interactions are reduced. Increasing dielectric loss becomes the reason for decreasing energy storage efficiency [Table 1]. Limiting ourselves to modest concentration loading is necessary.^63^ Wider applications of piezoelectric devices are made possible by the logistical action of fillers and polymers in PVDF-based composites and NF. Currently, lead-free ceramics, lead-based perovskite, metal oxides, carbon-based additives, organic–inorganic materials, and other materials are included as integrated fillers.^64^

**Table 1:** Performance of PVDF-Based Composite Nanogenerators with Different Fillers

s. no.	Material	technique/NG type	energy stored density/efficiency (representative)	*V*_oc_/*I*_sc_	references
1	ZnO (nanowires)	PENG (single-NW and arrays)	0.9 mW/cm^3^, 17 to 30%.	5.6 V/--	^75,76^
2	BaTiO_3_ (BTO)	PENG, composites (BTO/PDMS, BTO nanofibers)	0.1841 μW	2.67 V and current of 261.40 nA	^77^
3	BiFeO_3_ (BFO) with Sr doped	PENG/hybrid (piezo + ferro/photocoupled)	7.29 μJ within 500 s /∼0.31 μW cm^–2^	6.35 V and 0.64 μA	^78^
4	K_0_._5_Na_0_._5_NbO_3_ (KNN)	PENG (lead-free perovskite)	---------	54.1 V and 29.4 μA	^79^
5	PZT (Pb(Zr,Ti)O_3_)	PENG (thin films)	17.5 mW/cm^2^ at 200 MΩ	200 V and current of 150 μA/cm2	^80^
6	ZnSnO_3_/ZnSnO (zinc stannate)	PENG, PENG–composites	230 μW·cm^–2^	120 V, 13 μA	^81^
7	PVDF and MoS2-PVDF/PDMS	electrospinning, solution casting/TENG, HNG	··········	130 V, 35.3 V/12 μA, 20.8 μA	^70^,^71^
8	2DSnO_2_ /PVDF	hydrothermal/solution casting PSNG	6.25 μAcm^–2^/16.3%	42 V	^72^
9	(BiFeO_3_) (BaTiO_3_) PVDF	sol–gel route/electrospinning MPENG	··········	83 V/1.62 μA	^73^
10	ZnO/PVDF	solution casting	6.624 μW	6.9 V/0.96 μA	^74^
11	rGO/PVDF	electrospinning/in situ reduction PENG	··········	16 V/700 nA	^63^
12	ZnFe_2_O_4_/PVDF	hydrothermal/drop casting PENG	7.64 mJ cm^–3^/77.2% (ZF-r)	–39.10 V/–51.4 μA	^64^
13	(ZnO) (ZnSnO_3_) PVDF	hydrothermal/drop casting HNG	134.98 μJ cm^–3^/62.52%	···	^65^
14	BaTiO_3_-La/ PVDF-TrFE	hydrothermal/electrospinning TENG	87.3 μ Cm^–2^/···	···.	^66^
15	BaTiO_3_ /PVDF	solution casting/casting	4.12 J cm^–3^/63.2%	···	^67^
16	BaSrTiO_3_/PVDF	electrospinning/solution blowing PENG	··········	12 V/···	^68^
17	(BaCa)(ZrTi)O_3_ with PVP	sol–gel/tape casting by hot press	88.2 kJ cm^–3^/···	23 V/94 μA	^69^
18	silk fibroin	TENG/PENG/hybrid (films, electrospun fibers)	828.8 mW m^–2^	63.0 V, and a current of 2.4 μA	^82^
19	chitosan	TENG/bio-TENG (films, membranes)	-----------	∼0.7–242 V; ∼0.6–30 μA	^93–119^
20	Carrageenan	TENG (CS,SE mode)	--------	17 V–347.5 V	^125–128^
21	gelatin/fish gelatin	TENG (films, hydrogels)	------------	∼17–400 V/0.35 μA– 50 μA	^120–124^
22	Alginate	TENG/stretchable biointerfaces (hydrogels)	----------	3V-288 V/ 8.7 μA	^83–92^
23	hydroxyapatite (HAp)	PENG/composites (e.g., HA/PLLA composites for bone interface)	5 μW	∼3.4 mV/ 0.35 mA	^129^
24	polylactic lactic acid (PLLA)	TENG (biodegradable TENG layers), PENG composites	--------	45 V/9 μA	^130^
25	polycaprolactone (PCL)	TENG/implantable scaffold-integrated harvesters	---------	30 V/0.45 mA·m^–2^	^131^
26	cellulose acetate	TENG (paper-based and nanopaper devices)	352 μW	170 V/0.08 μA	^132^
27	graphene/2D materials	hybrid electrodes/active layers for TENG/PENG		3.0 V/250 nA cm^–2^	^133^
28	carbon nanotubes (CNTs)	conductive electrodes, composite fillers in PENG/TENG	37.8 mW m^–2^	180 V and 0.8 μA	^134^
29	MXene-PDMS	TENG electrodes/dielectric enhancement layers	19.6%.	145 V and 27 μA	^135^
30	BNT (Bi_0_._5_Na_0_._5_TiO_3_)	flexible PENG	3.95 mW m^–2^	22 V/140 nA	^136^
31	NaNbO_3_–Bi_0_._5_Na_0_._5_TiO_3_ composite	ferroelectric composite (energy storage)	2.464 J/cm^3^, efficiency 85.8%	—	^137^
32	PVA/PDAP/CNT	SR(1)-skin	-	70–95 V/0.89–1.24 mA m^–2^	^138^
33	CS(2)/Ag NWs/Cu	PDMS-skin	-	174→218 V/25→34.4 mA m^–2^	^139^
34	PSGP(3)	PSGP-PDMS	-	12 V/0.50 mA m^–2^	^140^
35	PAM/HEC/LiCl	SR-skin	-	285 V/17.2 mA m^–2^	^141^
36	P(MEA-IBA)/LiTFSI	VHB-latex	-	3 V/0.75 mA m^–2^	^142^
37	cellulose/NaCl	VHB-glove	-	187 V/0.57 mA m^–2^	^143^
38	PVA/PDAP/graphene	PDMS/CNT-Cu	-	69 −132 V/---	^144^
39	PAM/PVA/NaCl	ecoflex-skin	-	90–245 V/2.6–6.2 mA m^–2^	^145^

Table 1 presents a detailed comparative overview of essential materials utilized in nanogenerators (NGs), emphasizing their associated processes, output characteristics, and standard performance metrics. A distinct differentiation exists between ceramic-based piezoelectric nanogenerators (PENGs) and polymer- or biopolymer-based triboelectric nanogenerators (TENGs). Conventional inorganic materials like ZnO, BaTiO_3_ (BTO), and BiFeO_3_ (BFO) demonstrate moderate to high voltage outputs and energy densities ranging from microwatts per cubic centimeter to several milli-watts per cubic centimeter, attributed to their robust ferroelectric and piezoelectric interaction. Lead-free perovskites, including NaNbO_3_, and Bi_0_._5_Na_0_._5_TiO_3_ (BNT), have surfaced as effective, environmentally friendly alternatives to PZT, attaining similar energy densities ∼1–3 mW cm^–3^ and open-circuit voltages ranging from 22 V, with efficiencies reaching 8% via domain calignment and compositional modification. In contrast, biocompatible materials such as silk fibroin, chitosan, alginate, carrageenan, and hydroxyapatite exhibit comparatively higher energy conversion rates, owing to their flexibility, biodegradability, and nontoxicity. Hybrid composites, including MXene–polymer, graphene–PDMS, and hydrogel-based materials, exhibit the benefit of integrating mechanical strength with exceptional charge transfer, resulting in improved *V*_oc_ values and sustained output stability.

## 5 Next-Generation Self-Powered Sensors for Human Health Applications

Next-generation self-powered sensors represent a significant evolution beyond conventional sensing platforms by integrating energy harvesting, signal transduction, and data communication within a single miniaturized system. Unlike traditional sensors that depend on external power sources or bulky batteries, next-generation devices are capable of autonomously operating through ambient energy conversion from mechanical motion, body heat, or biofluids. This self-sustained operation not only reduces maintenance and replacement issues but also ensures uninterrupted, long-term monitoring—critical for wearable and implantable biomedical applications.^146–148^

The fundamental distinction between existing sensors and their next-generation counterparts lies in their material and structural innovations. Emerging energy-harvesting materials such as piezoelectric, triboelectric, and pyroelectric nanostructures allow simultaneous sensing and power generation, effectively reducing system complexity and energy consumption.^149,150^ Moreover, the incorporation of flexible, stretchable substrates enhances biocompatibility and mechanical conformity with human skin and tissues, ensuring comfort and reliability during prolonged use.^151^

Another defining feature is the integration of these self-powered sensors with artificial intelligence (AI) and the internet of medical things (IoMT) frameworks, which enable real-time physiological data collection, adaptive feedback, and predictive healthcare analytics.^152,153^ Such systems can monitor critical parameters—including heart rate, body temperature, respiration rate, glucose levels, and neural signals—with high precision, supporting the transition toward personalized and preventive medicine.^154^ The potential impact of next-generation self-powered sensors extends beyond continuous health monitoring to encompass remote diagnostics, rehabilitation tracking, and early disease detection. Their self-sustaining nature aligns with the global pursuit of sustainable healthcare technologies, minimizing electronic waste and dependence on disposable power units. Consequently, these devices are anticipated to play a pivotal role in developing smart, adaptive, and eco-friendly biomedical systems that seamlessly integrate with the human body, advancing the frontier of digital healthcare and precision medicine.^155–157^

Similarly, wearable biosensors have attracted significant attention in recent years due to their potential to deliver real-time, continuous health monitoring and enable personalized therapy strategies.^158,159^ These sensors are particularly useful in tracking physiological and biochemical changes, offering insights into both acute and chronic health conditions. A large proportion of molecular biosensors rely on electrochemical transduction mechanisms, including amperometry, potentiometry, differential pulse voltammetry (DPV), and impedance-based sensing modes. These electrochemical techniques provide high sensitivity, selectivity, and rapid response times, which are critical for early disease detection and ongoing health assessment.^160,161^

Additionally, electrochemical biosensors are highly compatible with flexible substrates and miniaturized circuits, making them suitable for integration into wearable and implantable platforms. They can detect a wide range of biomarkers, such as glucose, lactate, electrolytes, and other metabolic indicators. Various self-sufficient sensors, such as TENGs and PENGs, have been incorporated to drive these biosensors without the need for external power sources. These sensors can be configured into both implantable and wearable formats, enhancing user comfort and broadening their applicability in health monitoring systems, as illustrated in Figure 5.^162–164^

**Figure 5:**
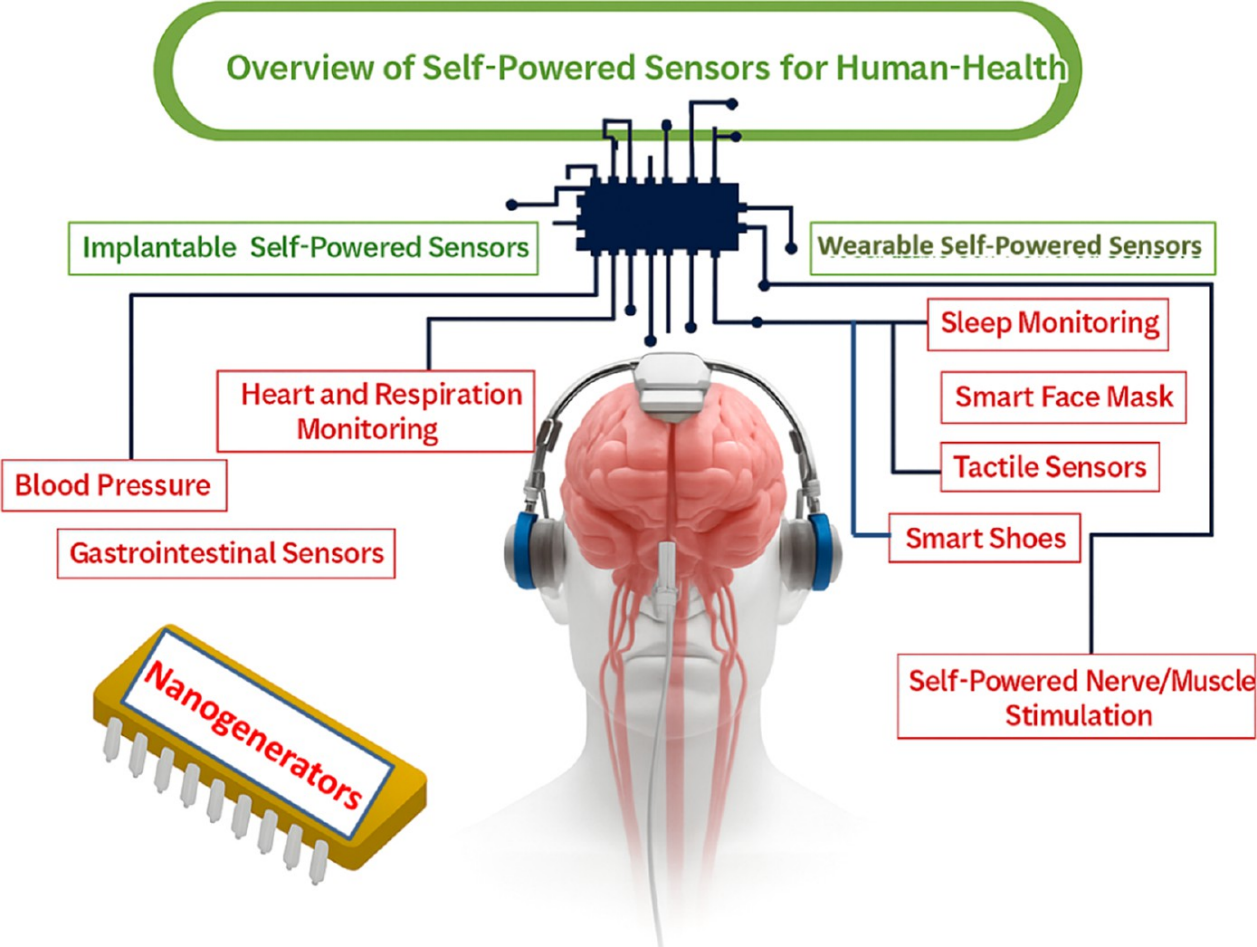
Next-generation self-powered sensors for human healthcare. Reproduced with permission from ref 164. Copyright 2020 Nature Reviews Materials.

## 6 Nanogenerators for Biomedical Applications

In the medical profession, implanting micromedical devices is often necessary to treat several sick organs. Lead-free, biocompatible PVDF material is an excellent option for energy harvesters in contrast to lead piezoelectric ceramic materials that may be poisonous to living things. Naturally, the cardiovascular system and interior joints are the best sources of persistent vibration energy, which has excellent potential for use in implanted energy harvesters.^165^ The potential of implanted biomedical devices (IBDs) to revolutionize the biomedical sector is the reason for the growing interest in these devices. The last several years have seen the emergence of IBDs, which greatly improve and extend human life, making it easier to live. A number of IBDs have been effectively used in in vivo settings, most notably prolonging the useful life of heart pacemakers by over 10 years. Even with these developments, there are still issues with battery-operated IBDs. Traditional batteries have a low energy density, a bulky and stiff construction, limited capacity, and hazardous components that add weight. Furthermore, patients face hazards, discomfort, and financial hardship when they must replace or remove batteries after a few years. The progress of self-powered Implantable Biomedical Devices that can capture surrounding and widespread bioenergy is essential to resolving these problems and minimizing the disadvantages of conventional battery-powered systems.

Within the field of bioenergy, the most prevalent, yet sometimes disregarded, source of energy in the natural world is biomechanical energy, which is produced by minute motions of internal body organs. The creation of implanted triboelectric nanogenerators (iTENGs) and implantable piezoelectric nanogenerators (iPENGs) is the result of recent technical developments. The utilization of biomechanical energy derived from internal body-organ motions by these devices offers a novel approach to addressing the sustainability issues associated with a conventional power supply in implanted devices. Biomechanical energy transforms to electrical energy in the case of TENGs, and contact electricity and the induction of electrostatic charge are coupled between moving triboelectric layers. When compared to batteries and other energy generation techniques, iPENGs and iTENGs have several benefits: they are more affordable, smaller, more biocompatible, and flexible, have a long-lasting power source, and are very sustainable. Because they can transform biomechanical energy into electricity, iPENGs and iTENGs have great potential as self-powered implanted devices for physiological sensing and therapy. Notwithstanding the general interest in them, particular difficulties must be addressed for effective implementation. To stop biofluid loss, which can reduce device performance, materials that are biocompatible for containment are being developed. Furthermore, for devices to be miniaturized and implanted into tight areas within organs and tissues, materials that are exceedingly sensitive and react to little mechanical motion must be developed. An overview of current developments in iPENGs and iTENGs is given in this paper, with an emphasis on uses in therapy, sensing, and energy harvesting and storage.^166^

Nanogenerators, particularly those based on piezoelectric and triboelectric mechanisms, have opened new frontiers in biomedical science by enabling self-sufficient devices that harvest biomechanical energy from the human body. These energy-harvesting systems support a wide range of uses, from implantable medical scaffolds to wearable health monitors and therapeutic patches. Below is a consolidated overview of their major biomedical uses.

### 6.1 Implantable and Tissue-Regenerative Nanogenerators

#### 6.1.1 Cardiovascular Scaffolds

Nanogenerators integrated within or attached to cardiovascular scaffolds convert natural body movements, such as cardiac pulsation and vessel deformation, into continuous electrical outputs. These generated microcurrents are distributed locally within the scaffold, maintaining a stable and consistent electrical environment. Such continuous power generation ensures reliable operation of the scaffold without external energy sources. Since cardiovascular tissues inherently respond to electrical impulses, the nanogenerator’s output naturally complements the existing electrical activity, creating a self-sustained and responsive system that enhances overall device performance and functionality.^167^ As a result, biomedical intelligent electronic devices have been established such as artificial pacemakers and ECG monitoring. These devices were initially large and heavy, external to the body, had a limited lifespan, and required significant surgical risk on the part of the patient to replace. Figure 5 describes the development of NGs for cardiovascular implant applications during the past 10 years from single thin wires to wireless interconnected devices. Nanogenerators embedded in cardiac patches or vascular scaffolds can stimulate cardiac tissue and power implantable biosensors, aiding in heart repair and monitoring.

To diagnose and treat heart disease and track the efficacy of treatment, cardiac sensing entails obtaining and evaluating data about heart function. Traditional cardiac sensing devices are intrusive because they often need batteries for electricity and cable connections to provide data to an external device. These gadgets have the potential to worsen patients’ discomfort and infection risks. Because of this, several studies in the past few years have shown the use of iTENG and iPENG, which let implanted devices work on their own, solving the problem that comes with traditional cardiac sensing devices. For cardiac monitoring, Zhao et al. have created a no-spacer TENG.^163^ Compared with a traditional TENG, the no-spacer TENG shows greater displacement and a more consistent stress–strain distribution with the exact same applied pressure. A Sprague–Dawley rat’s heart has a no spacer TENG implanted to detect breathing and cardiac disturbances. It has a 99.73% accuracy rate in heart rate monitoring and can pick up on subtle cardiac movements that an electrocardiogram cannot pick up on.^168,169^ Implantable devices that contain leakage-proof constructions and thin, flexible sheets have been employed to ensure optimal in vivo performance of iTENGs and to promote effective energy harvesting. A complete circuit is shown in Figure 6, which illustrates a self-powered pacemaker that is powered by the rat’s breathing and consists of a rectifier and a capacitor [171].^171^

**Figure 6:**
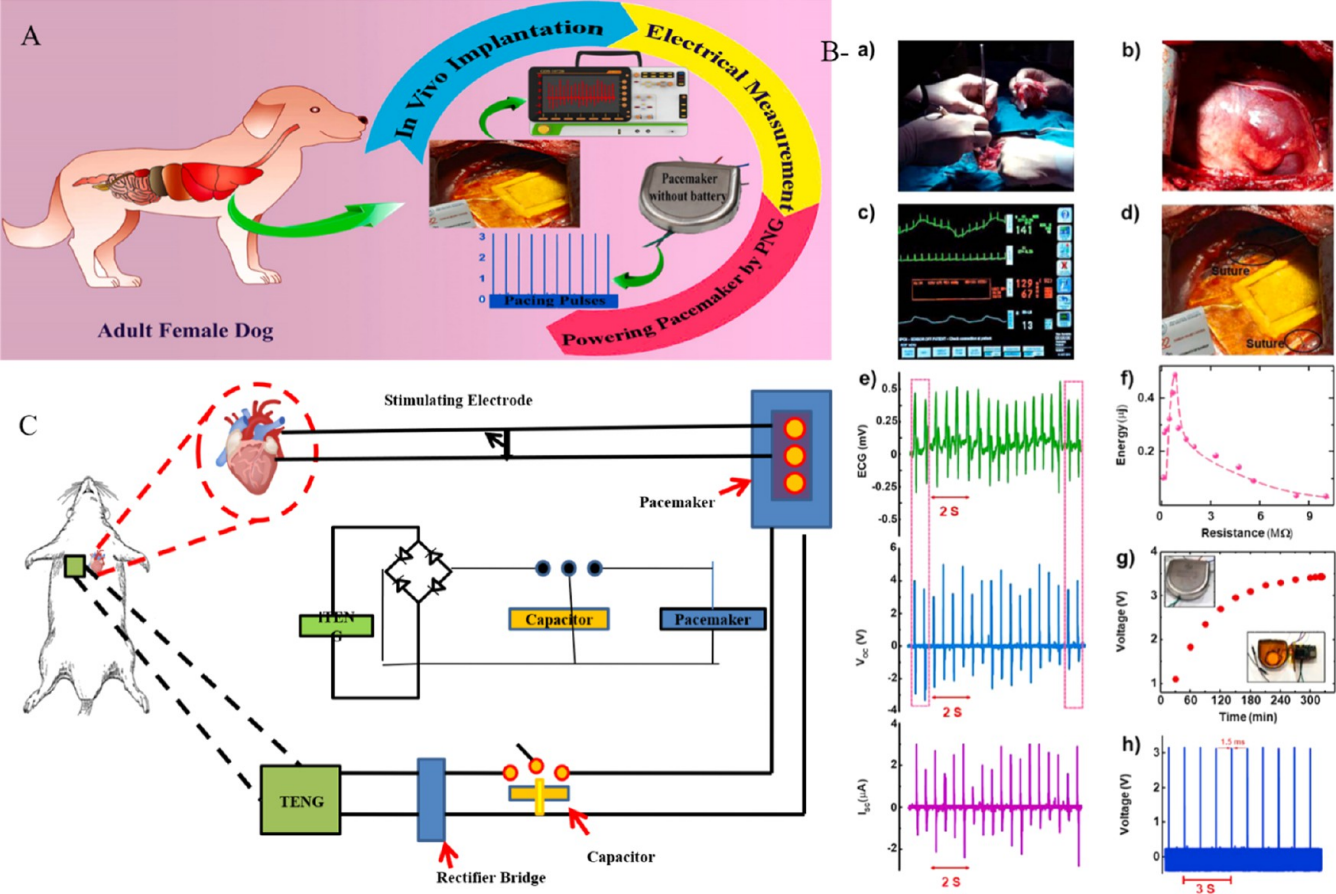
(A) Diagram illustrating a self-powered pacemaker in Adult Female Dog and their implantation circuitry. Reproduced with permission from ref 171. Copyright 2021 Nano Energy. (B) Pacemaker that runs on its own power. (a) The procedure of surgery in animal experiments. (b) A picture of the heart. (c) Blood pressure and ECG data captured concurrently. (d) The PENG sutured on the LV’s lateral wall facing the epicardium. (e) The PNG’s short circuit current, matching ECG signal, and in vivo open circuit voltage. (f) Using biomechanical energy harvesting of cardiac movements, the produced energy of PNG was assessed over a variety of load resistances. (g) The charging curve of a PNG-charged 100 μ F capacitor. The images of a viable pacemaker and the same device after the lithium battery is removed are displayed in the top-left and bottom-right insets, respectively. (h) Pacing pulses produced by the PNG-powered pacemaker. Reproduced with permission from ref 170. Copyright 2021 Nano Energy. (C) Diagram illustrating a self-powered pacemaker that is powered by the rat’s breathing and consists of a rectifier and capacitor. Reproduced with permission from ref 171. Copyright 2021 Nano Energy.

Similarly, Azimi et al. reported lead-based ceramic-PENGs, which are hazardous and vulnerable to fatigue cracks, inflicting injury to the patients. Additionally, films based on PVDF-TrFE were created as cardiac energy harvesters. Here, researchers demonstrate a battery-free heart pacemaker that uses the left ventricle’s cardiac movements to produce energy using a flexible and biocompatible piezoelectric polymer-based nanogenerator (PNG). Poly­(vinylidene fluoride) (PVDF) composite nanofibers and a hybrid nanofiller consisting of reduced graphene oxide (rGO) and zinc oxide (ZnO) make up the PNG. The composite nanofiber is designed to provide a lot of power. An optimized PNG implanted in vivo can effectively harvest 0.487 μJ from each pulse, which is suitably more than the human heart’s pacing threshold energy. An optimized PNG implanted in vivo can effectively harvest 0.487 μJ from each pulse, which is conveniently more than the human heart’s pacing threshold energy. The polymer-based PENGs are among the promising options for self-sufficient biomedical implants, as evidenced by the successful demonstration of a self-sufficient pacemaker.^170,171^

#### 6.1.2 Bone Tissue Engineering

Bone defects can occur due to various causes such as trauma, infections, or bone tumors, posing a major challenge in bone tissue engineering and regenerative research.^172–174^ To overcome these limitations, nanogenerators integrated into bone grafts or scaffolds have emerged as an effective strategy to convert mechanical stresses generated during natural body movements or rehabilitation into localized electric fields. These self-generated fields provide continuous internal stimulation that promotes mineral deposition and supports structural regeneration without the need for external power sources. The on-demand, activity-driven electrical output of nanogenerators not only accelerates bone defect healing and enhances bone mineral density but also synergizes effectively with osteoconductive and osteoinductive materials, resulting in a more adaptive and sustainable approach for bone tissue engineering applications. In this context, bioelectric potentials have been shown to play a critical role in the natural bone healing process, making the use of electroactive materials a promising approach for bone repair.^174–178^ Among these, biopiezoelectric materials with excellent biocompatibility have attracted significant attention for their ability to stimulate bone regeneration. These include inorganic materials such as potassium-sodium niobite and barium titanate as well as organic polymers like poly­(vinylidene fluoride) (PVDF), poly-l-lactic acid (PLLA), and polyhydroxybutyrate (PHB). Efforts to enhance the piezoelectric properties of these materials have led to the development of advanced electrically stimulated bone repair scaffolds. As early as 2001, Chen et al. proposed a composite material combining hydroxyapatite and barium titanate leveraging the intrinsic piezoelectric nature of bone. Their study demonstrated the effectiveness of this composite in both in vitro and in vivo (canine) models.^179^ In addition, Heng et al. provided a comprehensive review of degenerative bone diseases and highlighted the therapeutic potential of various natural and synthetic electroactive biomaterials. They explored the underlying mechanistic pathways through which these materials promote bone regeneration, including enhanced osteogenesis and angiogenesis, anti-inflammatory properties, inhibition of osteoclastogenesis, and antibacterial effects.^180,181^

The field has seen rapid advancement since then, particularly in the design of piezoelectric ceramics for orthopedic use. Liu et al. developed BTO/PA12 composite scaffolds by incorporating barium titanate into polyamide 12 (PA12) using laser sintering techniques.^182^ To overcome issues related to BTO agglomeration in polymers, they utilized polydopamine (PDA), which introduces functional groups (amino and hydroxyl) capable of forming strong hydrogen bonds. This modification significantly improved the electrical output of the scaffold and enhanced proliferation, cell adhesion, and osteogenic differentiation.

Further, Liu et al. examined the structural advantages of 3D-printed composite scaffolds and emphasized the importance of achieving piezoelectric polarization and bioelectric coupling for effective bone graft substitutes. However, challenges remain in optimizing scaffold architecture to induce consistent regenerative outcomes.^183^

In another study, Giovanna Strangis et al. developed piezoelectric nanocomposite scaffolds using polyhydroxybutyrate (PHB) and barium titanate (BaTiO_3_) nanoparticles for vascularized bone tissue engineering which are illustrated in Figure 7. By varying BaTiO_3_ content (5–20 wt %), they observed significant improvements in mechanical strength and piezoelectric performance, with a maximum *d*_31_ value of 37 pm/V at 20% BaTiO_3_. The nanocomposites were fabricated into 3D-printed porous scaffolds with suitable pore sizes (0.60–0.77 mm) and demonstrated excellent mechanical stability and minimal degradation (∼4%) over 8 weeks in saline. This study highlights the potential of BaTiO_3_/PHB scaffolds as multifunctional platforms for bone regeneration through structural support and bioelectric stimulation.^185^

**Figure 7:**
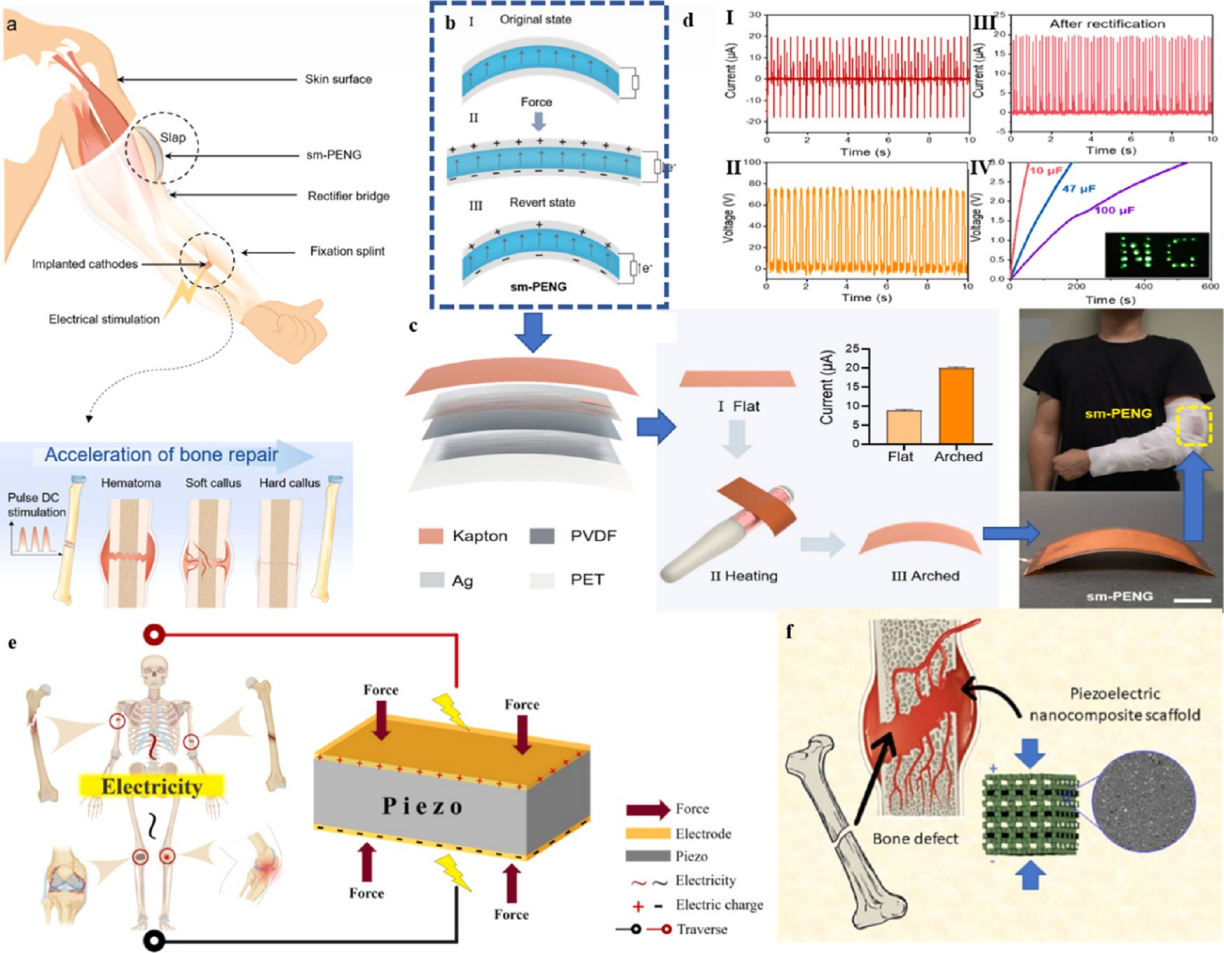
The schematic diagram of self-powered electrical stimulation for bone repair. (a,b) Schematic of the integrated self-powered pulsed-DC device based on sm-PENG and fracture fixation splint for bone repair in vivo. Electrical stimulation accelerates bone repair. (c) Preparation and characterization of sm-PENG with schematic structure of the sm-PENG and Kapton film was processed by thermo forming technology to realize arch structure and the short-circuit current of the device before and after thermo forming. This also provides integration with a fixation splint. Scale bar: 1 cm. (d­(i)) Short-circuit current of the sm-PENG. (ii) The short-circuit current after rectification. (iii) The open-circuit voltage of the sm-PENG. (iv) The charging profile of three different specifications of the sm-PENG that could directly power 23 LEDs, Reproduced with permission from ref 183. Copyright 2021Nanoenergy. (e) Application Framework of Piezoelectric Nanogenerators in Bone Healing and Repair. (f) Piezoelectric nanocomposite used for bone defect. Reproduced with permission from ref 184. Copyright 2023 Material Today Communication.

### 6.2 Skin-Integrated and Wearable Nanogenerators

#### 6.2.1 Hair Regeneration Devices

Hair loss remains a common physiological and psychological concern, significantly affecting the self-image and quality of life. Recent advancements have shown that nanogenerators can offer an innovative self-powered approach to hair regeneration by converting natural head movements or slight skin deformations to low-level electrical outputs. These generated microcurrents help modulate the follicular microenvironment and stimulate dermal papilla cell activity, thereby promoting the transition of hair follicles into the anagen (growth) phase. Unlike conventional wired or battery-operated systems, nanogenerator-based wearable patches provide localized, continuous, and low-intensity electrical stimulation during routine activities. Their lightweight, flexible, and unobtrusive design makes them suitable for long-term use, offering an efficient and sustainable strategy for noninvasive hair regeneration applications.Conventional solutions, such as surgical hair transplantation, offer only partial success and come with procedural limitations.^186^ Recent studies indicate that electrical stimulation, particularly in the form of pulsed electric fields, can effectively enhance hair follicle activity and promote regeneration. With the growing integration of TENG technology into regenerative medicine, TENG-driven electrical stimulation is emerging as a noninvasive and promising approach for hair regrowth.

In a pioneering study, Yao et al. 2019 developed a wearable TENG-based electrical stimulation device (ESD) designed to induce hair regeneration. Its design is illustrated in Figure 8. When applied to Sprague–Dawley (SD) rats, the ESD maintained a stable voltage output of 320 mV and functional integrity over 28 days. The alternating electric field generated by the device significantly improved hair growth, achieving an average length of 15.4 ± 2.1 mm within three weeks—demonstrating superior efficacy compared to traditional treatments.^187^ Although promising, further optimization of stimulation parameters and clinical validation are essential to confirm the long-term safety and therapeutic effectiveness of this technology.

**Figure 8:**
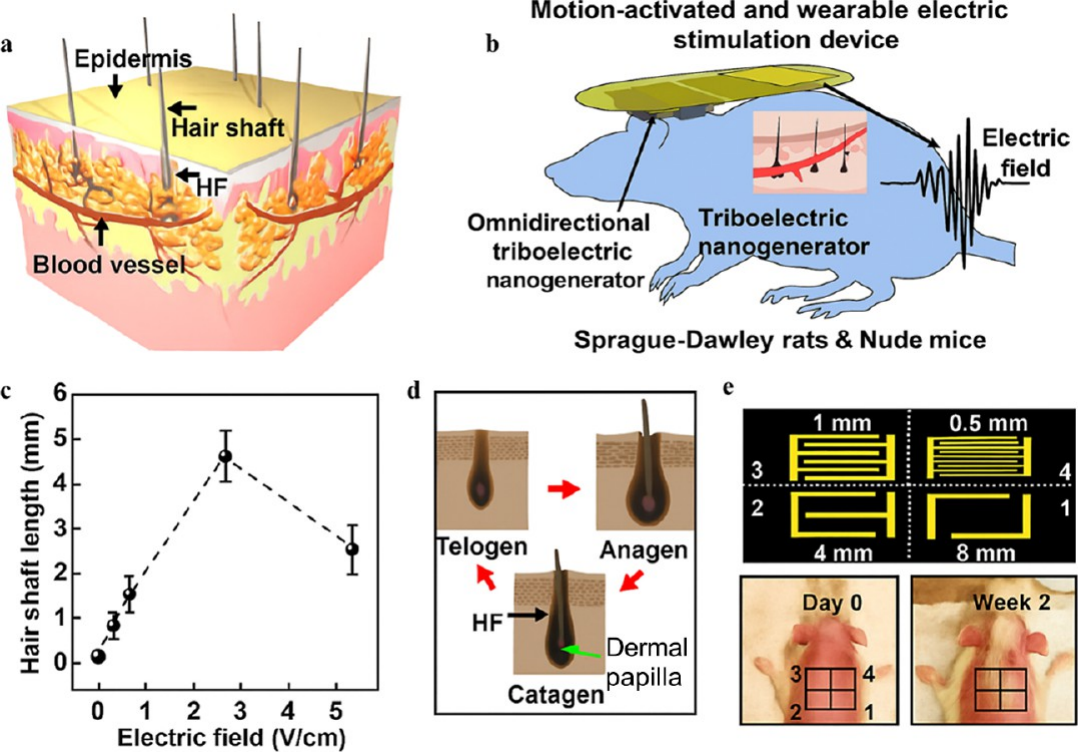
Hair regeneration effect of SD rats under the stimulation of the *m*-ESD: (a) Schematic illustration of HFs in skin. (b) Wearable TENG-based electrical stimulation device (TENG-ESD) designed to induce hair regeneration. (c) Histomorphological schematic of the hair cycle including anagen, catagen, and telogen stages. (d) Schematic diagram of a series of interdigitated electrodes (1–4) with different gap widths. (e) Optical images of the rat with removed hair (day 0, left) and after 2 week treatment (right). Reproduced with permission from ref 187. Copyright 2019 ACS Nano.

#### 6.2.2 Drug-Loaded Patches

Nanogenerator-based drug-loaded patches have emerged as an innovative self-powered platform for controlled and on-demand drug delivery. These systems utilize the mechanical energy generated from body movements or skin deformation to produce localized electric signals, which, in turn, regulate the release of drugs from electroresponsive polymers or carrier matrices. The electrical output from the nanogenerator can modulate diffusion rates, activate ion migration, or induce structural changes in the patch material, enabling precise control over the dosage and release timing. This self-sustained mechanism eliminates the need for external power supplies or complex circuitry, making the system compact and energy-efficient. The major advantages include real-time, activity-triggered drug release, enhanced therapeutic efficiency, and improved patient compliance due to its wearable, noninvasive, and battery-free design. Overall, nanogenerator-integrated drug patches represent a promising approach for personalized and responsive drug delivery systems.^188^

A notable innovation came in 2023 when Wang et al. introduced a microneedle-based TENG patch (TENG-MN) specifically engineered for deep tumor therapy. The patch demonstrated enhanced drug permeability and therapeutic effectiveness in melanoma-bearing mice, validated through histological tissue analysis.^189^ The system represents a high-efficiency, minimally invasive platform suitable for future applications in diseases such as diabetes and cancer. Despite its promise, the long-term stability and patient comfort associated with such hydrogel-TENG hybrids remain areas for improvement. Additionally, Du et al. developed an integrated, rectifier-free triboelectric nanogenerator (TENG) patch featuring surface-engineered electrodes for simultaneous drug loading and release and localized electric field generation to accelerate wound healing. The electrode was fabricated via in situ growth of magnesium–aluminum layered double hydroxide (Mg–Al LDH) nanosheets on aluminum foil (LDH@Al), followed by minocycline incorporation to form a minocycline-loaded electrode (MLDH@Al). The arch-shaped TENG patch, composed of MLDH@Al, polytetrafluoroethylene (PTFE), and a flexible polymer substrate, exhibited excellent skin conformability and multifunctionality as shown in Figure 9. In vitro and in vivo results showed controlled electrical stimulation and sustained minocycline release, effectively inhibiting *Staphylococcus aureus* (∼96.7%) and promoting rapid tissue regeneration, achieving complete healing of infected full-thickness wounds in mice within 10 days. Furthermore, the antibacterial activity of low-intensity alternating current (AC) local electric fields (LIEFs) generated by the TENG was attributed to time-accumulated electrical breakdown and electrochemically induced hydrogen peroxide (H_2_O_2_) generation. This study represents the first application of an AC LIEF-based TENG for in vivo infected wound treatment, providing a promising direction for self-powered and multifunctional healthcare electronics.^190^

**Figure 9:**
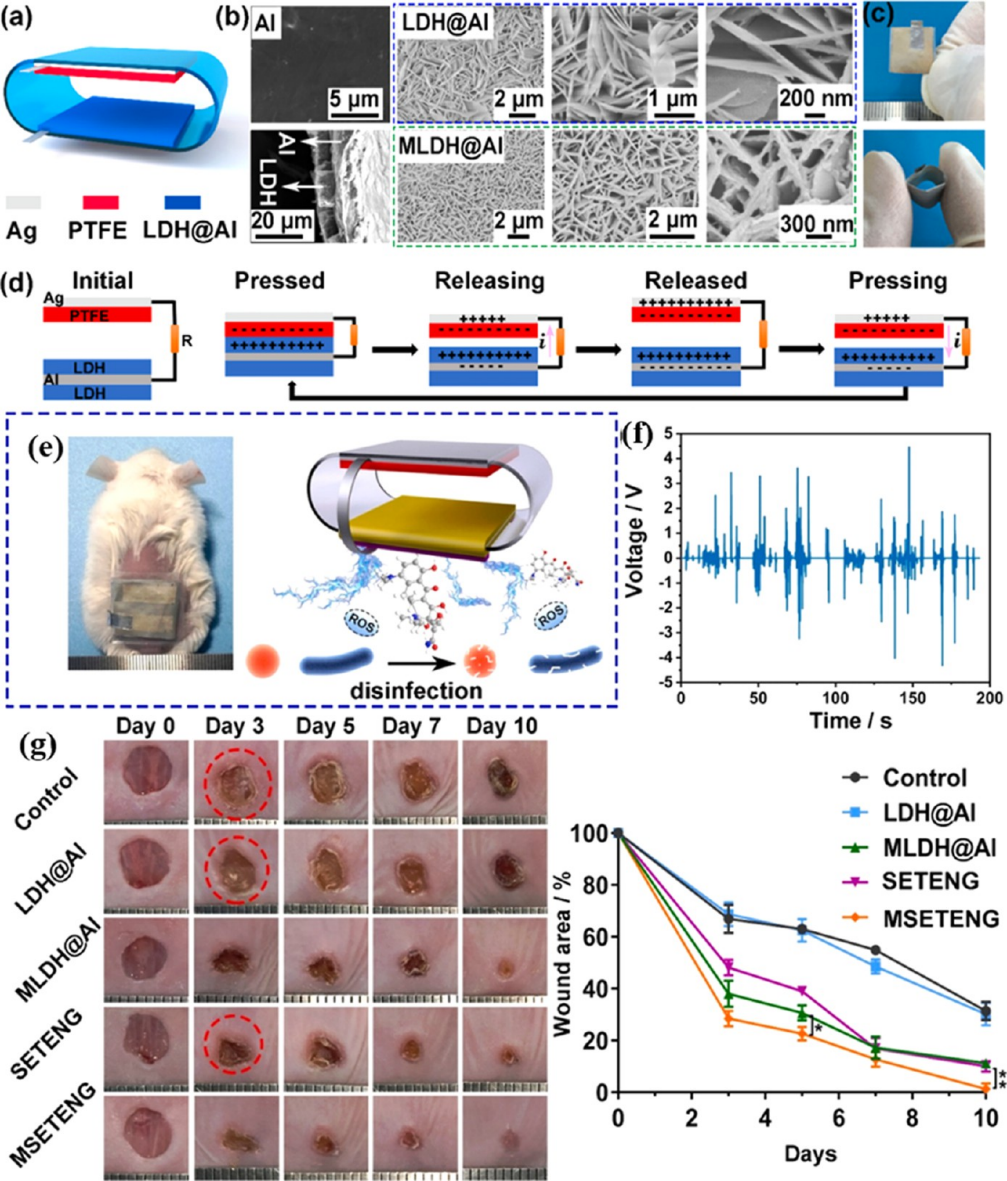
Schematic illustration of the surface-engineered TENG and drug loading. (a) Structural design of the TENG with surface-engineered LDH@Al as the electrode and friction layer. (b) SEM images of Al foil, the LDH@Al film, and the LDH@Al film with minocycline (MLDH@Al). (c) Optical photographs of SETENG. Top panel: top-view image of the TENG patch. Bottom panel: squeezed TENG patch. (d) Working principle of the SETENG. Effects of surface-engineered TENG patches on the in vivo wounds infected by *S. aureus*. (e) Photograph of the mouse wearing the MSETENG patch on infected wounds in the back and the proposed mechanism for promoting infected wound healing. (f) Output voltages of MSETENG patch induced by the mouse’s motion. (g) Representative photographs of the skin wound at different periods after wearing different patches and the wound area quantification. Reproduced with permission from ref 190. Copyright 2023 ACS Applied Materials & Interfaces.

#### 6.2.3 Electronic/Energy Skin (E-Skin)

Energy skin (e-skin) represents an emerging frontier in wearable nanogenerator (NG)-based technology engineered to replicate the tactile and sensory functions of human skin while operating entirely without external power sources. NGs integrated within flexible e-skin frameworks enable self-powered, multimodal sensing—including detection of pressure, strain, shear, and temperature—through synergistic piezoelectric, triboelectric, or hybrid mechanisms. The mechanical deformations produced by body movements or environmental interactions are directly converted into electrical signals, which can be harnessed for both real-time tactile mapping and energy harvesting to drive local signal processing or wireless data transmission. This self-sustained operation eliminates dependence on external batteries, offering continuous monitoring with high spatial resolution, mechanical flexibility, and long-term durability. When coupled with AI-driven data interpretation, NG-based e-skin opens new possibilities for gesture recognition, prosthetic feedback systems, and personalized physiological monitoring, marking a transformative advancement in next-generation wearable interfaces (Figure 10).^191^ It holds transformative potential across domains like prosthetics, robotics, and real-time health monitoring.^192^ Out of NGs, TENG plays a pivotal role in e-skin by converting mechanical inputs—such as touch, motion, and pressure—into usable electrical energy, enabling the skin to sense and respond dynamically.^193^ As in 2018, Liu et al. engineered hydrogel-based TENGs exhibiting excellent hydration retention and stability, proving their suitability for long-term e-skin applications.^194^ Li et al. 2023, developed a dual-network ionic hydrogel e-skin (PXS) combining a PXS-Mn^+^/LiCl primary cell and TENG system with a capacitor. This device successfully detected complex human movements, from joint flexion to facial expressions and even sound vibrations.^195^ Beyond hydrogels, Rana et al. designed a composite-based CDL-TENG using Co-functionalized nanocomposites (Co-NPC). Their system demonstrated an excellent triboelectric output (21 V, 9.85 μA/m^2^) and sensitivity to humidity and acceleration, highlighting its potential for obstacle detection and smart human-machine interaction.^196^ Additionally, Zhou et al. depicted the structural design, working mechanism, and multifunctional applications of a flexible and self-powered electronic skin (e-skin) based on an ultrastretchable triboelectric nanogenerator (STENG). The multilayered TPU/AgNWs/rGO configuration provides remarkable elasticity (up to 200% strain) and mechanical durability, ensuring stable charge generation under repeated deformation. As illustrated in the mechanism schematic (Figure 10a–e), the synergistic effect between the conductive AgNWs/rGO layers and the stretchable TPU substrate enables efficient triboelectric charge transfer, resulting in a high open-circuit voltage (∼202 V) and power density (∼6 mW/m^2^). The e-skin also exhibits an excellent pressure sensitivity (78.4 kPa^–1^) and an ultrafast response time (1.4 ms), demonstrating a superior tactile sensing performance (Figure 10).^197^

**Figure 10:**
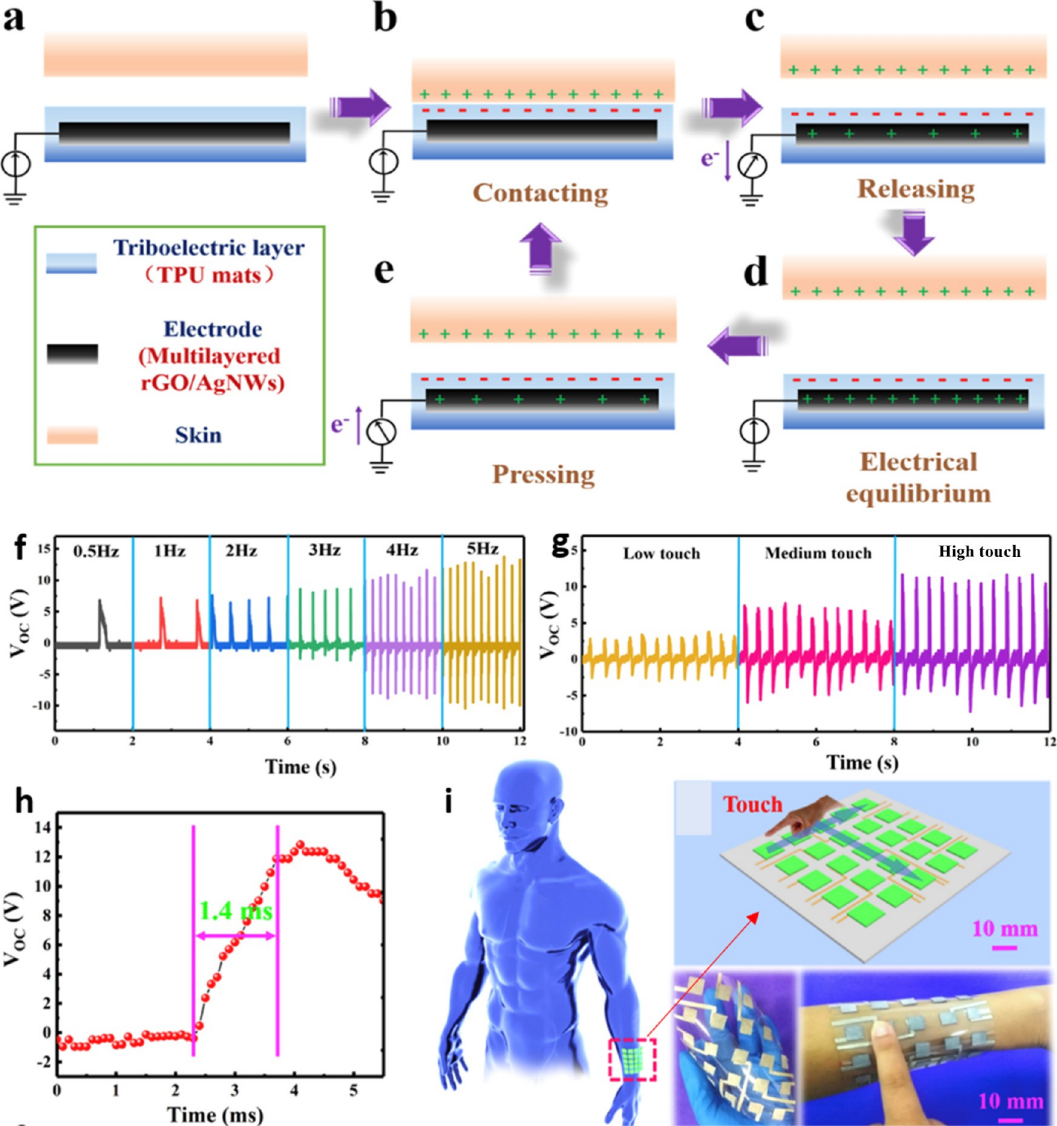
(a–e) Working mechanism diagram of STENG-based e-skin. Applications of the self-powered e-skin in tactile sensing and energy harvesting. *V*_OC_ of the single e-skin with different frequencies (f) and strengths (g) of finger touch. (h) The response time of the e-skin is under 5 Hz. (i) Photographs of the e-skin array attached to the forearm state and the connection of the flexible tactile sensing e-skin array with 5 × 5 pixels. Reproduced with permission from ref 197. Copyright 2020 Nano Energy.

#### 6.2.4 Breathable and Sweat-Absorbing/-Resistant Devices

Breathable and sweat-absorbing or sweat-resistant nanogenerator (NG)-based devices represent a crucial advancement in wearable electronics, addressing the comfort, durability, and reliability challenges of long-term skin contact applications. By integrating porous, hydrophobic–hydrophilic balanced substrates such as nanofiber membranes or breathable elastomers, these devices allow efficient air and moisture exchange while maintaining robust mechanical flexibility and output stability. The triboelectric or piezoelectric layers are engineered to resist performance degradation caused by perspiration, ensuring consistent energy harvesting from natural body movements, even under humid or high-sweat conditions. Additionally, moisture-wicking or superhydrophobic surface designs prevent salt accumulation and electrical shorting, extending the device lifespan. Such breathable NG systems not only enhance user comfort but also enable continuous power generation and biosignal sensing during exercise, rehabilitation, or daily wear, marking a vital step toward practical, skin-friendly self-powered wearable.^197,198^ To address this, researchers have developed moisture-wicking, breathable materials capable of operating under humid or strenuous conditions. Li et al. created a bionic superhydrophobic vertical contact-separation TENG inspired by a lotus leaf architecture. The structure ensured self-cleaning and high-performance operation even in extreme humidity, making it ideal for athletic monitoring and outdoor use.^199^ A bioinspired sweat-resistant wearable triboelectric nanogenerator (BSRW-TENG) was created to mitigate sweat interference during exercise and facilitate real-time motion monitoring. The apparatus consists of two superhydrophobic and self-cleaning triboelectric layers—elastic resin and polydimethylsiloxane (PDMS)—exhibiting hierarchical micro/nanostructures modeled after lotus leaves. These structures enhanced the electrical output 2-fold and provided the gadget with significant resistance to contamination and dampness. Following saltwater exposure, the BSRW-TENG exhibited consistent performance but a flat TENG saw a 41% reduction in production attributable to salt deposition. Despite an increase in relative humidity from 10% to 80%, the output of the BSRW-TENG decreased by just 11%, in contrast to a 54% reduction observed in the flat equivalent. It demonstrated resilience under whole surface contamination and water-spray conditions. The BSRW-TENG proficiently tracked several workouts, such as dumbbell curls, leg curls, and running, sustaining consistent output pre- and postsweating, hence indicating its potential as an economical, sweat-resistant wearable sensor for fitness and sports performance assessment.^199^ In a parallel study, Zhi et al. introduced a directional moisture-wicking e-skin (DMWES) with a porous gradient design and asymmetric wettability. The system effectively conducted sweat away from the skin, improving pulse detection, gait analysis, and voice recognition in real-world human testing.^200^

Similarly, Li et al. developed a Janus nanofiber textile inspired by plant vascular systems. The textile’s hydrophilic side drew sweat via capillary action, while the hydrophobic side prevented moisture return, enhancing wearer comfort. This innovative approach opens the door to scalable production of breathable, self-sufficient e-textiles for health monitoring (Figure 11).^199^

**Figure 11:**
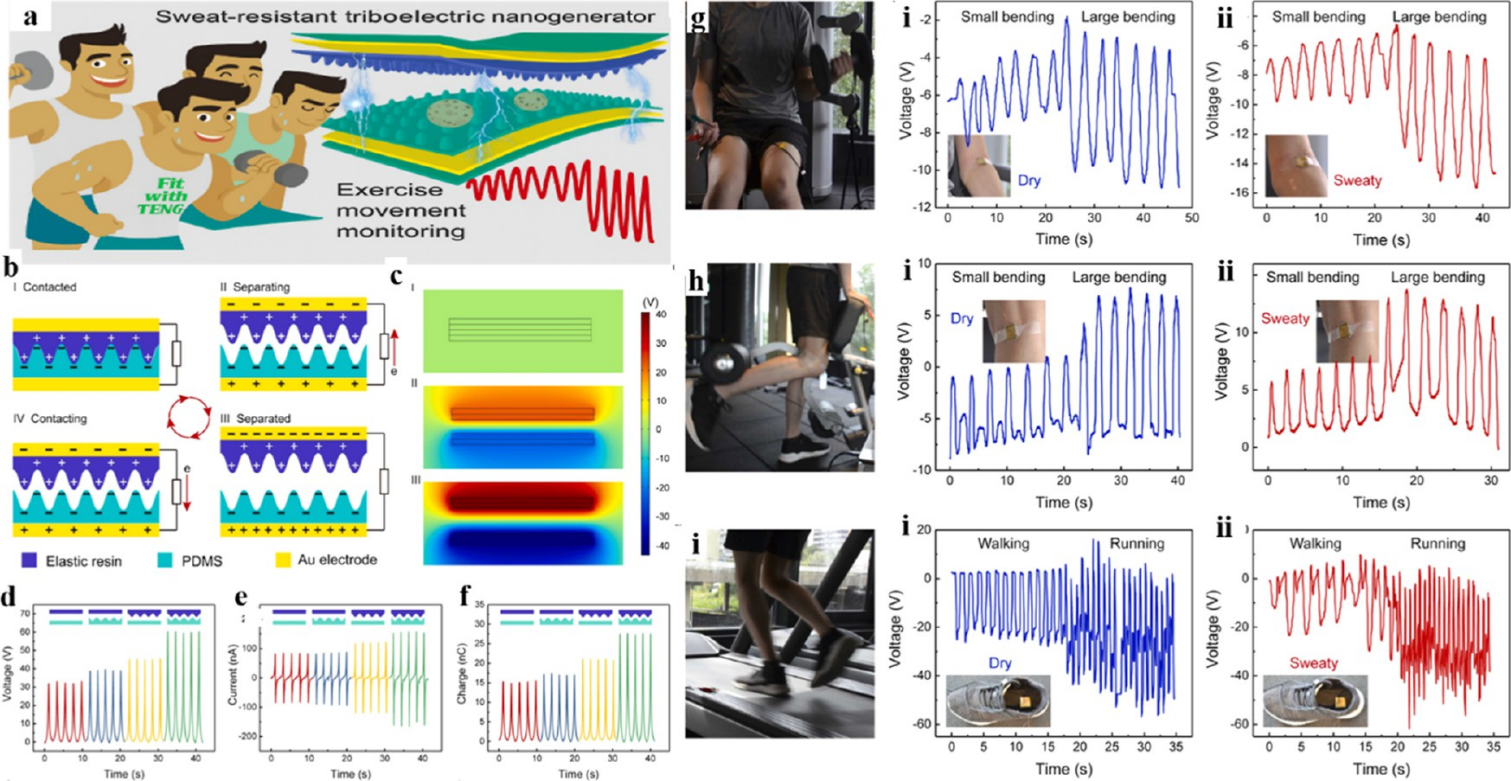
(a) Schematic diagram of sweat-resistant triboelectric nanogenerator (TENG). (b) Working principle of the BSRW-TENG. (c) Simulated electric potential distribution on the triboelectric surfaces during contact and separation. (d) Open-circuit voltage, (e) short-circuit current, and (f) transferred charges of TENGs with different triboelectric surfaces (flat resin and flat PDMS, flat resin and BH-PDMS, BH-resin and flat PDMS, and BH-resin and BH-PDMS). (g) Photo of the BSRW-TENG attached on the elbow joint for dumbbell biceps curl monitoring. Dumbbell biceps curl monitoring results (i) before and (ii) after sweating. (h) Photo of the BSRW-TENG attached on the knee joint for leg curl monitoring. Leg curl monitoring results (i) before and (ii) after sweating. (I) Photo of the BSRW-TENG attached on the sole for running monitoring. Running monitoring results (i) before and (ii) after sweating. Reproduced with permission from ref 199. Copyright 2022 Nano Energy.

#### 6.2.5 Hearing Aids

Hearing loss affects millions worldwide and often leads to reduced communication ability, social withdrawal, and cognitive decline.^201^ Traditional hearing aids, while effective, remain limited by battery dependence and maintenance needs. Recent advances in acoustic nanogenerators (NGs) based on triboelectric nanogenerator (TENG) technology have opened new pathways for self-powered hearing devices. These NGs can efficiently harvest ambient mechanical vibrations—such as environmental sounds, speaker-generated waves, or even subtle jaw movements—and convert them into electrical signals that either supplement or fully replace conventional batteries. In addition to power generation, NGs can function as self-powered acoustic sensors, enabling adaptive sound amplification and noise modulation without external energy sources. The key advantages include prolonged operational life, miniaturization of device components, reduced maintenance, and sustainable, continuous auditory monitoring, making them a promising solution for next-generation, energy-autonomous hearing aids.^201^ As in 2018, Guo et al. designed a triboelectroacoustic sensor (TAS) tailored for hearing aids and robotic auditory systems. With tunable resonance through geometrically optimized membranes, the device amplified specific frequency bands effectively, demonstrating adaptability for long-term wear.^202^ Building on this, Zheng et al. (2021) developed a BaTiO_3_/PVDF-TrFE acoustic harvester structured in a core–shell format for use as a cochlear implant prototype. Implanted in a model ear, the device successfully converted sound waves into electrical signals, aligning closely with the original audio inputs. This represents a critical step toward biocompatible, self-powered cochlear stimulation systems.^203^ Similarly, Mokhtari et al. reported that the transformation of sound vibrations into electrical signals is a vital function of cochlear hair cells (Figure 12), which unfortunately cannot regenerate once damaged, leading to irreversible hearing loss. To address this, a recent study explored piezoelectric filament—including PVDF, PVDF-BaTIO3, and PVDF-rGO as self-sufficient acoustic sensors that mimic hair cell function. These flexible filaments respond to sound frequencies ranging from 50 to 1000 Hz at sound pressure levels of 60–95 dB, achieving an acoustoelectric conversion efficiency of 3.25% and a high sensitivity of 117.5 mV (Pa·cm^2^)^−1^. Their cytocompatibility was validated through in vitro tests on inner ear-like cell lines, indicating their potential for future use in cochlear implants.^27^

**Figure 12:**
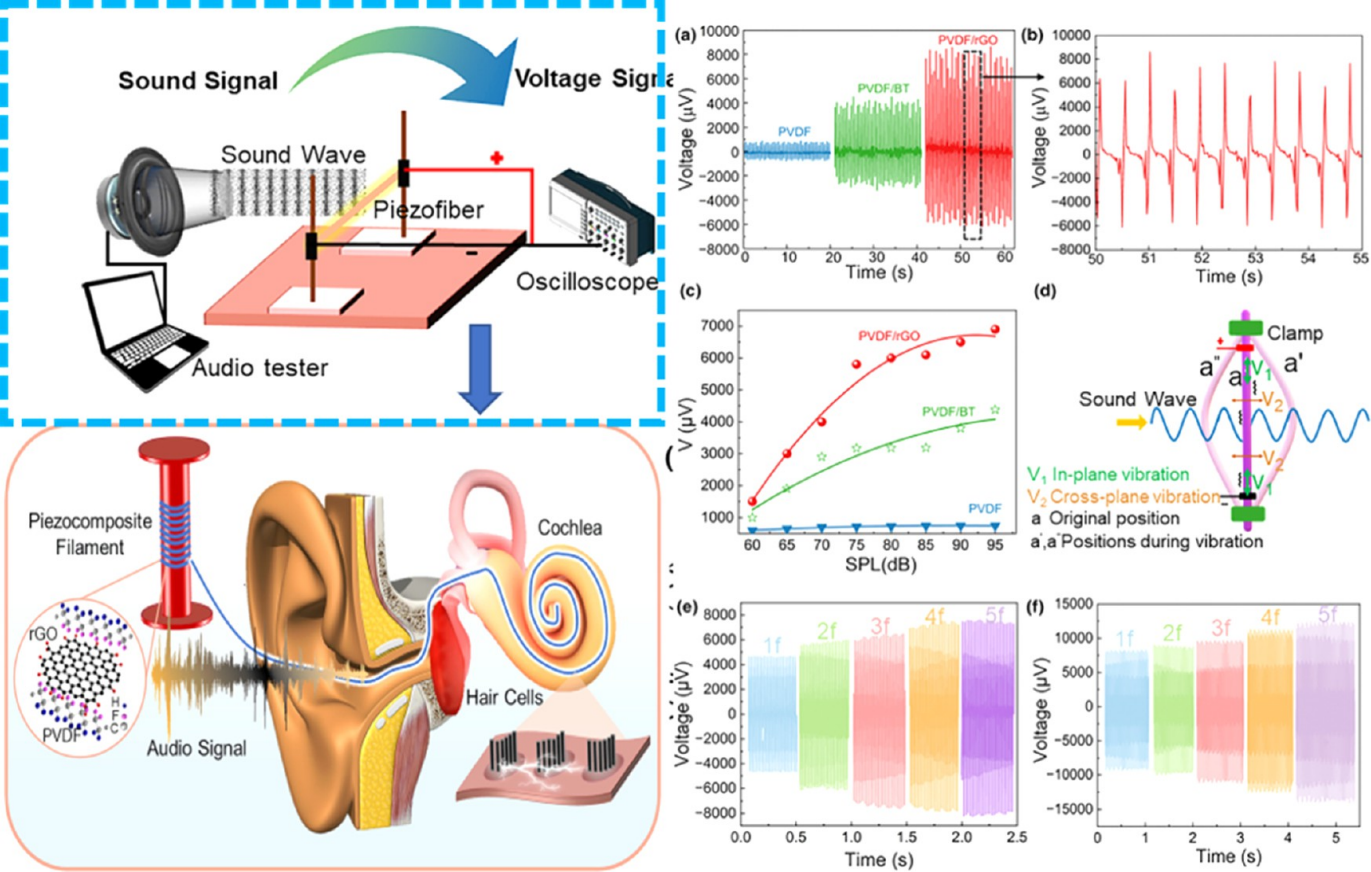
Schematic illustration of the setup for exposing filaments to sound waves and indicating piezoelectric nanocomposite filaments as artificial cochlear cells. (a) Maximum voltage outputs of piezo composite filaments at a frequency of 250 Hz and pressure of 95 dB. (b) The enlarged depiction of the open circuit voltage within the time frame of 50–55 s. (c) SPL versus voltage outputs at 250 Hz. (d) Proposed mechanism illustrating the effect of sound waves on piezoelectric conversion in nanocomposite fibers. Parallel connection of 1–5 nanocomposite filaments of (e) PVDF/BT and (f) PVDF/rGO at 250 Hz. BT, barium titanate; PVDF, poly­(vinylidene fluoride); SPL, sound pressure level; and rGO, reduced. Reproduced with permission from ref 27. Copyright 2025 Energy & Environmental Materials.

#### 6.2.6 Ligament Strain Sensors

Real-time monitoring of ligament strain can be achieved using nanogenerator (NG)-based sensors integrated into wearable braces or implanted anchors. These devices convert mechanical deformation of ligaments into self-generated electrical signals, enabling the continuous mapping of strain, loading cycles, and recovery progress without the need for external power sources. By providing immediate feedback, the sensors can alert users or clinicians when movements exceed safe thresholds, support rehabilitation by guiding activity, and even enable closed-loop systems, where detected strain triggers corrective actuation or targeted stimulation. In addition to rehabilitation applications, NG-based strain sensors offer a noninvasive method to assess ligament health, detect early signs of injury, and optimize personalized recovery strategies, all within a compact and fully self-powered platform. So, Sheng et al. published a paper special for describing an implanted silicone/osteogel fiber-helical sensor based on TENG for ligament strain monitoring,Figure 13.^205^ Silicone and spiral-twisted organogel fibers were used to construct the OFS-TENGs. A Cu wire was then joined to the organogel end; the spiral-twisted fibers were then placed inside a silicon tube and taken out of the tube, and both ends were covered with silicone. This gadget was fixed in the patellar joint of a rabbit knee to monitor knee ligament muscle tension and stretch. Ex vivo and in vivo tests show that the silicone/osteogel fiber-helical sensor based on the TENG is a very delicate, stable, and nontoxic device. Advance years, there has been an increased focus among researchers on the development of implanted sensors for monitoring ligament strain. These sensors hold the potential to assess and maintain the health of your ligaments and spot damage early.^205^ An implanted silicone/osteogel fiber-helical sensor based on a TENG (OFS-TENG) for measuring ligament strain was described in a recent study by Sheng et al., as shown in Figure 13.

**Figure 13:**
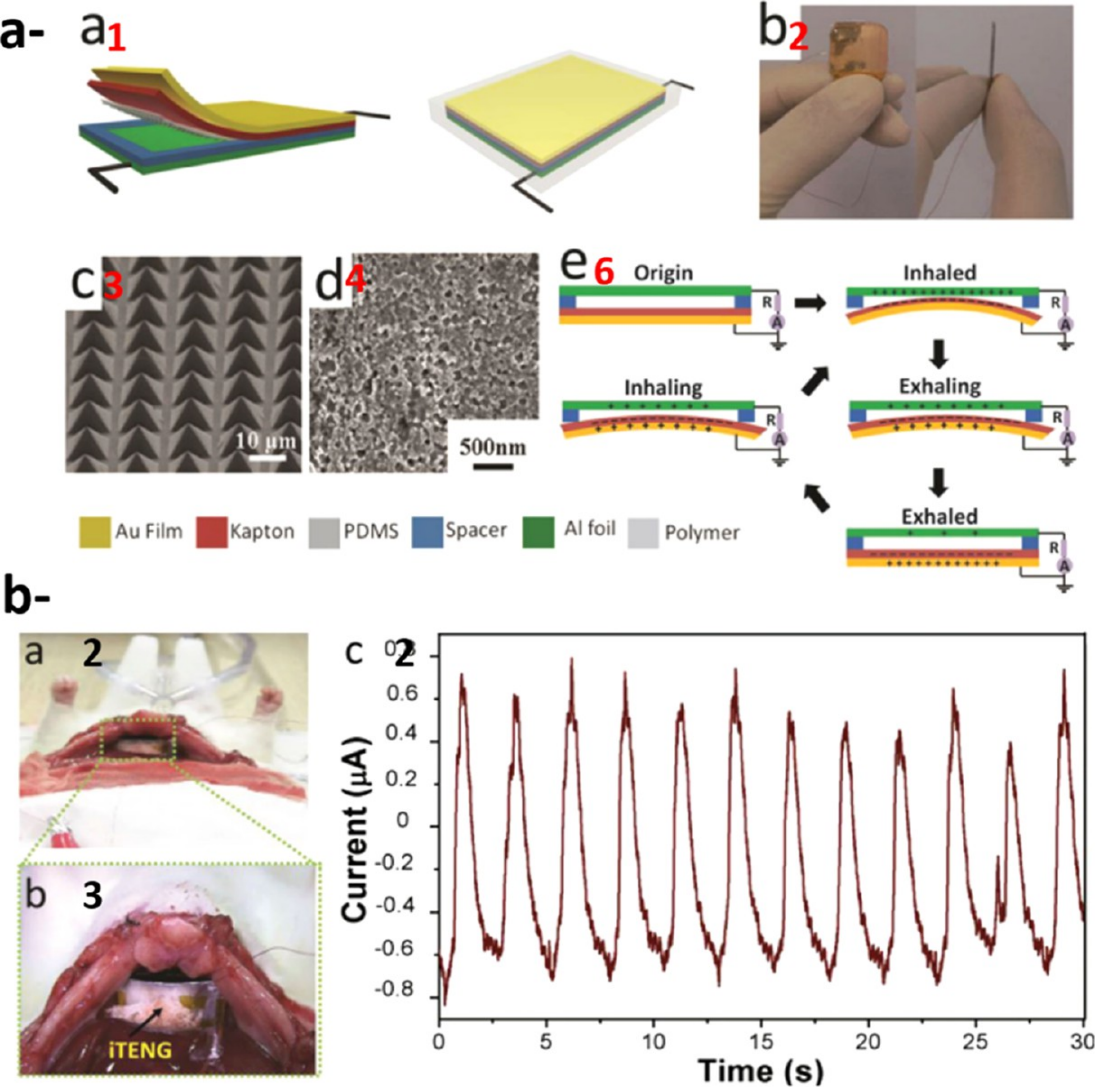
(a­(1)) Schematic of the multilayer TENG structure. (b(1)) Flexible TENG attached to skin, showing wearability. (c(1)) SEM image of the micropatterned PDMS surface (10 μm scale). (d(1)) SEM image of the porous triboelectric layer (500 nm scale). (e(1)) Working mechanism of TENG during breathing cycles. (b)­(a(2)–b(2)) iTENG implanted in an animal model showing placement and integration with muscle tissue. (c(2)) Real-time current output of TENG during breathing, showing periodic signals. Reproduced with permission from ref 205. Copyright 2022 ACS Nano.

#### 6.2.7 Bladder Sensors

An autonomous, self-powered bladder-assist device can be realized by using nanogenerator (NG)-based sensors that detect bladder volume through mechanical deformation or pressure changes. The NG converts these mechanical signals into electrical outputs, which can be processed to determine bladder fullness and trigger an actuation mechanism that assists urination. This self-sustained operation eliminates the need for external power sources, enabling continuous, real-time monitoring and intervention. Key benefits include autonomous and timely bladder emptying, reduced dependence on manual or catheter-based methods, enhanced patient comfort and independence, and a minimized risk of urinary retention-related complications. By integrating sensing and actuation in a single, self-powered platform, NG-based bladder devices offer a promising solution for individuals with neurogenic underactive bladders, providing responsive, safe, and energy-efficient urinary management. In a noteworthy development, Arab Hassani et al., 2018 devised a bistable actuator that demonstrates bladder discharge. This actuator is made of flexible PVC sheets and shaped-memory alloy (SMA) parts. The actuator exhibits full bladder voiding with repeated actuation, thanks to a bistable mode with compression and regeneration phases. With only 20 s of operation, this device can empty up to 78% of the bladder capacity in an anesthetized rat, demonstrating one of the greatest documented voiding capabilities. This actuator includes a TENG sensor built in to measure the degree of bladder fullness in individuals with underactive bladders who have impaired detrusor muscle and nerve functioning.^206^ The sensor-based actuator device that was created for this study has shown a lot of promise in helping bladders with weak muscular control and lost feeling. Future research will need to focus on creating SMAs with more rapid recovery times and larger power outputs because the current SMAs have a somewhat lengthy recovery period.

#### 6.2.8 Respiration-Driven Device

Nanogenerator (NG)-based respiration-driven devices can harvest energy from natural breathing motions by converting mechanical expansion and contraction of the chest or abdomen into electrical output. Typically, TENGs are attached to the thoracic or abdominal regions, where respiratory movements induce a periodic deformation of the device layers, generating continuous electrical signals. This self-powered mechanism can be used to drive wearable electronics, health monitoring sensors, and low-power biomedical devices without relying on external batteries. Key advantages include continuous energy harvesting from routine respiration, noninvasive integration, lightweight and flexible design, and the ability to power long-term physiological monitoring systems in a fully autonomous manner.^204^ Li et al. recently released a paper that provided a thorough explanation of the respiration-driven device. In particular, the device deforms and periodically moves, either laterally or vertically (CS and LS modes, respectively), as a result of the regular relaxation and contraction of muscles brought on by breathing. This produces a triboelectric output. An LS-mode TENG belt is placed over the chest, as shown in Figure 14. Breathing in causes the chest to expand and the belt to extend, causing the dielectric films to separate from one another. Exhaling causes the belt to loosen and the chest to compress, returning the films to their starting positions. The electrical output often synchronizes at a low frequency with the respiratory pattern in response to this kind of motion.^207^

**Figure 14:**
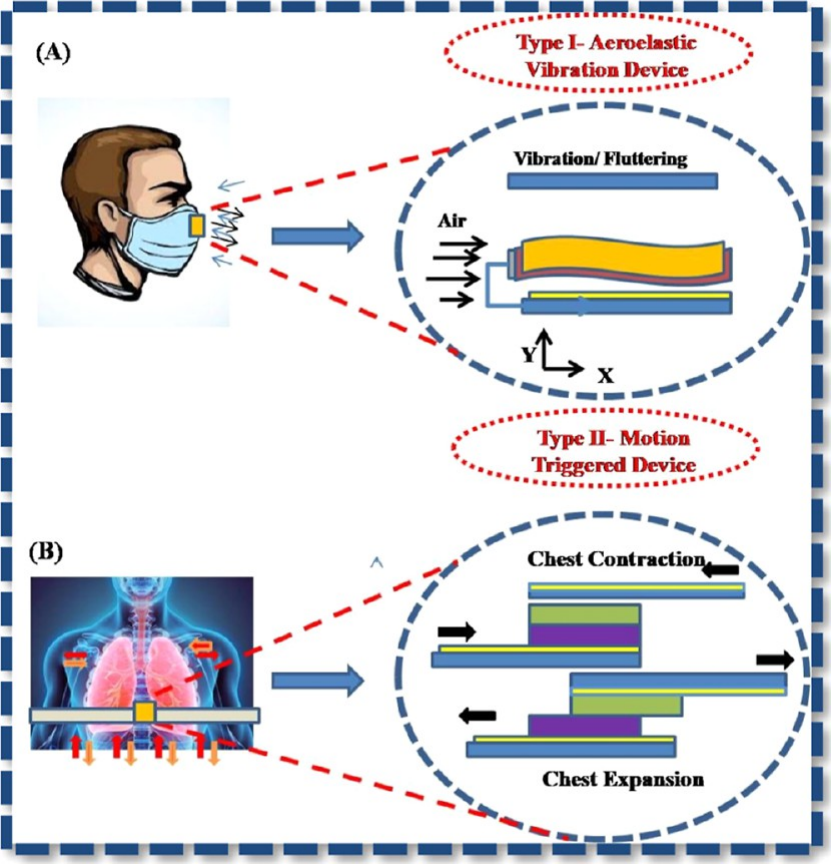
Schematics of the two primary varieties (a,b) of respiration-driven TENG, which extract energy from breathing at various points. Triboelectric nanogenerator or TENG. Reproduced with permission from ref 207. Copyright 2020 EcoMat.

#### 6.2.9 Eye Sensor

Nanogenerator (NG)-based eye sensors can harvest energy from subtle ocular movements, such as blinking or eyeball rotation, by converting these mechanical motions to electrical signals. Flexible triboelectric or piezoelectric layers integrated into eyewear or ocular patches detect the mechanical deformation caused by eye activity, generating self-powered outputs that can be used for real-time monitoring or control of assistive devices. This self-sustained operation eliminates the need for external batteries, enabling continuous, lightweight, and unobtrusive sensing. Key benefits include noninvasive monitoring of eye motion, potential applications in visual prosthetics or human-machine interfaces and energy-autonomous operation, making NG-based eye sensors suitable for long-term wearable or implantable use. For human-machine interfaces (HMIs), triboelectric nanogenerators exhibit significant promise as flexible motion transducers. The current study investigates the nonattached electrode-dielectric triboelectric sensor, a novel arrangement, and its use in a customized HMI to assist those with impairments in their everyday life. In this architecture, noncontact electrostatic induction creates a voltage in a different conductor as a result of the triboelectric interaction between the two moving parts. This makes it possible to use triboelectric and electrostatic coupling for near-field remote sensing. An Orbicularis Oculi muscle movement sensor has been created to track both voluntary and involuntary eye blinks using the nonattached electrode-dielectric triboelectric sensor sensing approach. To help those with mobility impairments, the novel transducer is incorporated into a portable HMI for hands-free computer cursor control. The developed gadget was also examined for use in monitoring driving behavior and as a hands-free remote control for cars and drones. Other benefits of the Non-Attached Electrode-Dielectric Triboelectric Sensor for detecting unusual motion dynamics have also been investigated with a PDMS-based eyelid movement sensor (Figure 15).^28^

**Figure 15:**
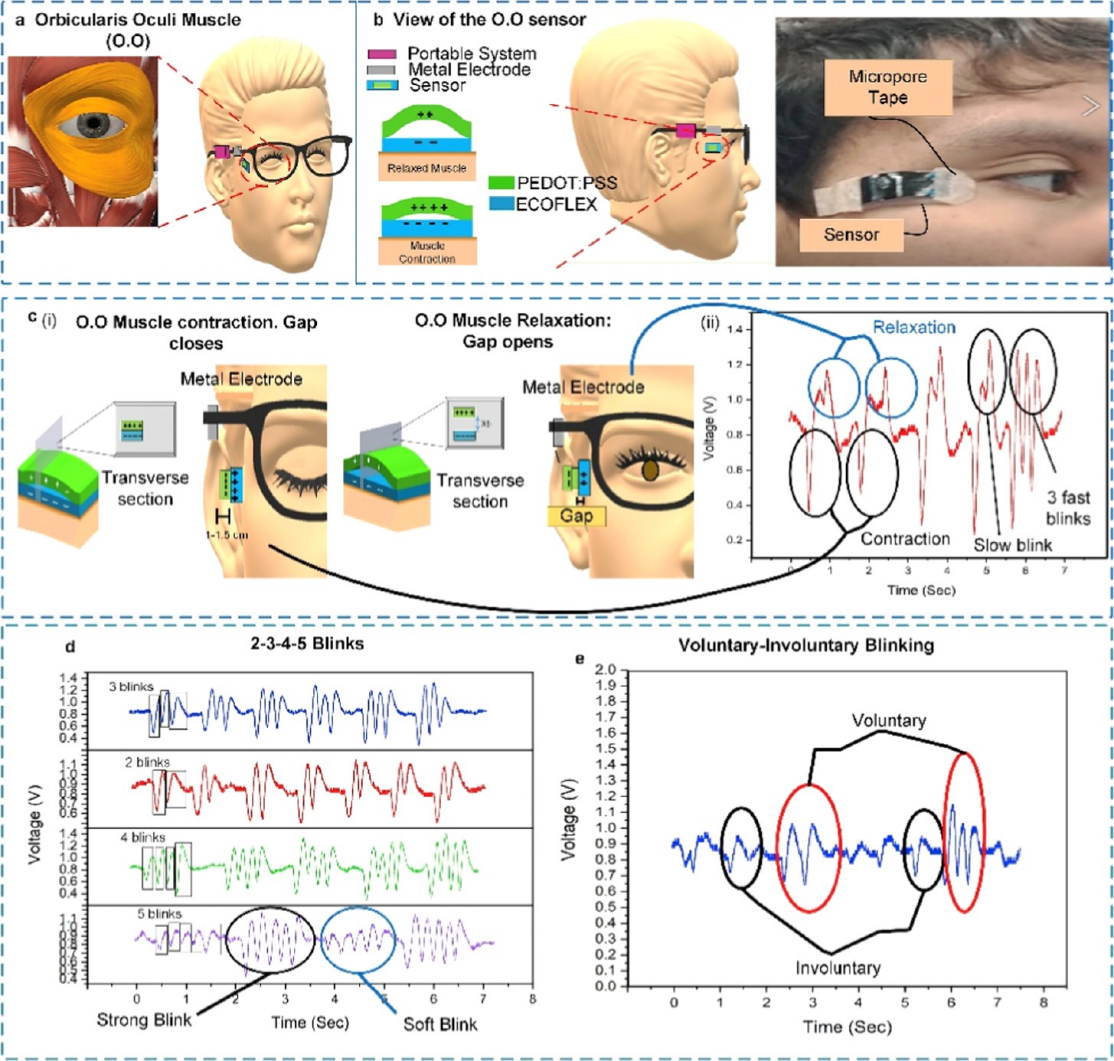
Design, positioning, and outcomes of eye movement sensors. (a) The area behind the eye is surrounded by the Orbicularis Oculi muscle. (b) Sensor positioning and overview. (c) (i) The muscle contracts and the sensor layers stretch when the eye is shut. Both the muscle and the sensor layers relax when the eye is opened. It shows the transverse section. (ii) Signals of contraction and relaxation. Blinking slowly and quickly. (d) The output signal from 2, 3, 4, and 5 consecutive blinks, both gentle and powerful. (e) Flickers, both involuntary and voluntary, Reproduced with permission from ref 28. Copyright 2020 Nano Energy.

#### 6.2.10 Wearable Application

Nanogenerator (NG)-based wearable sensors can capture a wide range of human motions and physiological signals by converting mechanical deformations into electrical outputs. When attached to joints such as the arm, elbow, or leg or integrated into footwear, NGs transduce bending, stretching, or foot pressure into measurable electrical signals, enabling real-time detection of movement patterns, gait, and posture. Similarly, when positioned near the chest, these sensors can harvest subtle respiratory- and heartbeat-induced motions to provide continuous physiological monitoring. The self-generated electrical signals can be processed directly or interfaced with microcontrollers, such as Arduino systems, to drive indicators or control devices, for example, activating LEDs to signal walking, running, or standing states.^209^ This self-powered approach eliminates the need for external batteries, allows continuous and autonomous sensing, and offers flexible integration into wearable formats, including smart shoes, clothing, or assistive devices such as smart chairs. Such NG-based wearables enable applications ranging from motion tracking and rehabilitation to personalized health monitoring and human-machine interface systems, all within a lightweight and fully autonomous platform. So, the TENG gadget was utilized in health-monitoring applications like heartbeat and breathing monitoring and to generate biomechanical energy from everyday human activities. The sensor tracked a variety of human body movements. A TENG that is both portable and flexible was created using a nanofiber film of PVDF doped with copper oxide 2 wt %, 4 wt %, 6 wt %, 8 wt %, and 10 wt % corresponding to PVDF content. Polyurethane (PU) was regarded as a counter-positive film and PVDF-CuO as a tribo-negative film for the TENG device fabrication.^207^ Lastly, real-time uses, including human mobility and health monitoring (heart rate and breathing), were showcased for the optimized device. The various uses of the PC-8/PU triboelectric sensor are listed in Figure 16. The sensor output electrical voltage during tapping, bending, twisting, and rolling is displayed in Figure 16a. In this instance, fast movements produced a voltage larger than that of slower movements, as seen in Figure 16b. More intriguingly, when employed as a theft monitor sensor, it is illustrated in Figure 16c. When the triboelectric sensor was placed in pants, a chair, and a shoe, Figure 16d–f shows that it was able to discriminate among various bodily actions, including walking, jumping, sitting, and kicking. The sensor was positioned beneath the chest in a prone posture, as seen in Figure 16g, to confirm the device used as a heartbeat monitor such as a health monitoring sensor. It generated secondary respiration peaks as well as primary heartbeat peaks.^208^

**Figure 16:**
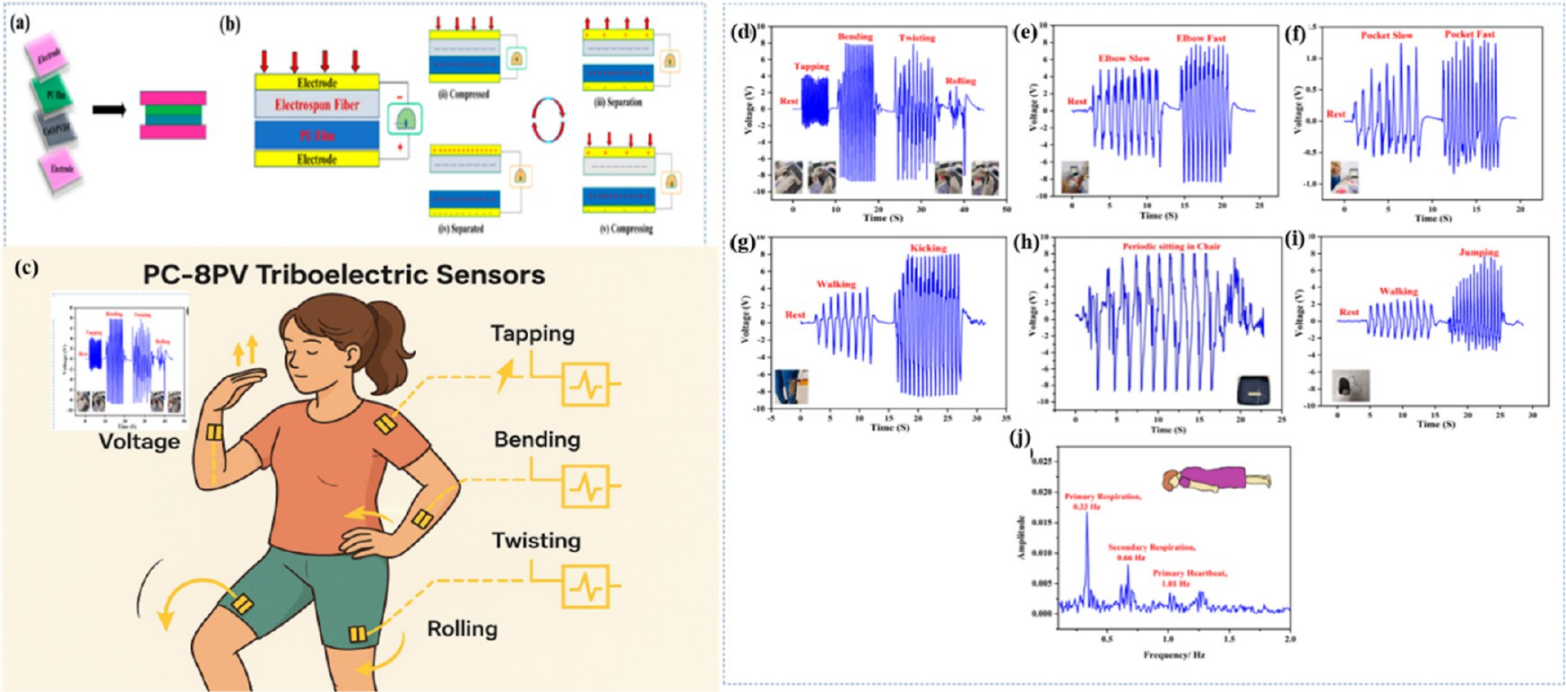
(a) Wearable applications of PC-8/PU TENGs include: graphical view of the TENG device; (b) diagram illustrating the charge-generating process of the PVDF–CuO TENG, (c) schematic diagram for all functions by using PC-8/PU, (d) detecting arm movements; (e) detecting elbow movements; (f) detecting leg movements; (g) detecting motion when the sensor is attached to a shoe; (h) uses for healthcare application with smart chairs; (i) detecting motion when the sensor is attached to a shoe; and (j) monitoring a person’s heartbeat and respiration when the sensor is placed close to the chest while they are in a prone position. Reproduced with permission from^208^. Copyright, Open access, MDPI publishing.

For instance, using no fragile or sensitive materials, Huang and Chung also described a three-layer TENG insole nanogenerator. It was made using an NBCC conductive layer and a microneedle PDMS dielectric layer. To ascertain the user’s condition, it was subsequently employed as a sensor in a self-powered human treading state detection system. Once the flexible and stable microstructure of the PDMS dielectric layer was demolded by using a CO2 laser, a PMMA mold was made to produce the microneedle surface structure and coat on the PET to withstand the form of the dielectric layer. The microneedle construction improved the microneedle-PDMS-TENG’s electrical output profile; the PDMS-TENG’s *V*_oc_ values with and without the microneedle were 129.2 and 54.6 V, respectively, representing a 237% increase. The *I*_sc_ values increased by 245% to 64.00 μA and 26.16 μA, respectively. The power values increased by 599% to 4.1 mW and 684 μW, respectively. It took 4 s for the PDMS-TENG to achieve 1.21 V and 3 s for the microneedle-PDMS-TENG to reach 1.75 V when charging the 1 μF capacitor. Regarding LED lighting, the PDMS-TENG could illuminate up to 90 LEDs, while the MN-PDMS-TENG could illuminate up to 120 LEDs (Figure 17). A nylon substrate and a conductive cloth were combined to create the NBCC conductive layer. The goal of this design was to retain comfort while guaranteeing the insole’s longevity under human striding. To maintain the optimal MN-PDMS-TENG stroke at 6 mm and guarantee that each step produced a full contact and separation movement, the three-layer TENG insole mechanism was designed as the NBCC, microneedle-PDMS-TENG, the nylon support layer, and the PE spacers. Because the mechanism did not contain any hard or brittle materials, the insole remained flexible and soft. Thirteen LEDs were illuminated by the 3-layer TENG insole, which produced a *V*_oc_ of 87.2 V. The 3-layer TENG insole’s force sensitivity, as determined by voltage and force data under various forces, was 0.07734 V/N, with a coefficient of determination of *R*_2_ = 0.91 under human stepping. *F* = 12.93 V – 92.10 was the function between force and *V*_oc_ that was determined. Three-layer TENG insoles were formed to create the self-powered human stepping state sensor system. These insoles were placed underneath the user’s front and back feet. The microcontroller (MCU) was an Arduino UNO, and the display devices were three sets of LEDs. The user can manage the devices, carrying out the related actions with different stepping states, such as running, walking, and standing, by using the Arduino UNO to calculate the difference in force data between the forefoot and back foot. This study illustrates the three-layer TENG insole’s potential for usage as a self-sufficient sensor and its use in sports rehabilitation and monitoring.^209^

**Figure 17:**
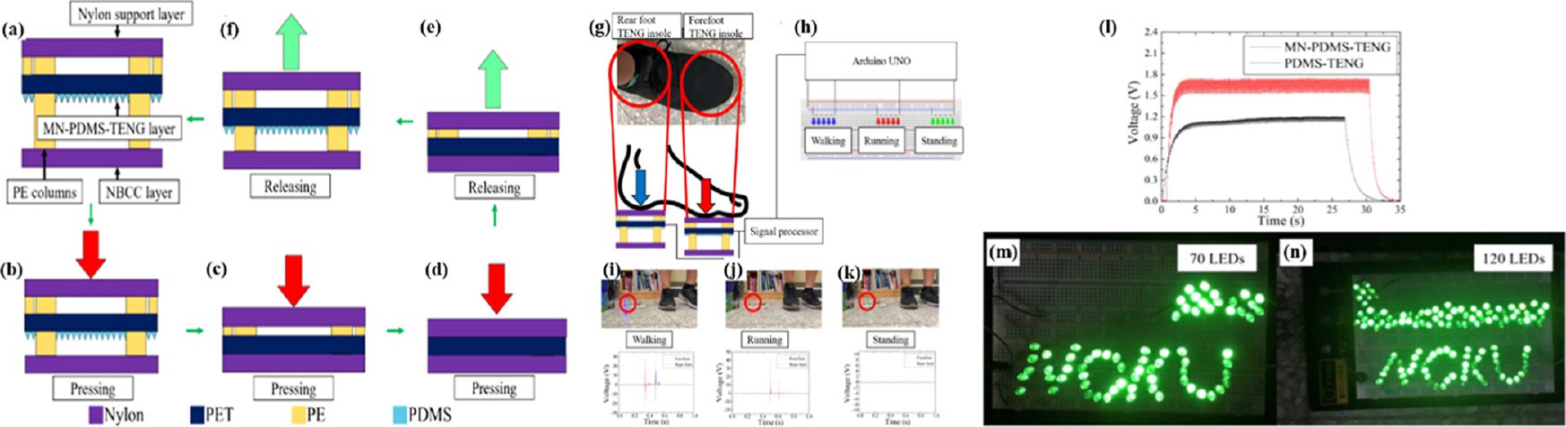
The working mechanism of the MN-PDMS-TENG insole involves a contact–separation process: (a) initially, all layers remain at rest; (b) under vertical stepping force, the PDMS-TENG and NBCC layers move closer; (c) they make full contact as the nylon support layer approaches the PDMS-TENG; (d) maximum compression causes all layers to contact completely; (e) upon release, the nylon layer separates first; and (f) finally, the NBCC layer detaches, restoring the system to its initial state. This periodic contact–separation generates an electrical output from human motion. Two three-layer TENG insoles were used as self-powered sensors (g), with signals processed by an Arduino UNO MCU (h) to drive LEDs: (i) blue for walking (<300 N force difference), (j) red for running (>300 N), and (k) green for standing (no step for 2 s). The energy-harvesting performance showed that (l) the PDMS-TENG charged a 1 μF capacitor to 1.21 V in 4 s. At the same time, the MN-PDMS-TENG reached 1.75 V in 3 s, lighting (m) 70 and (n) 120 LEDs, respectively, confirming its higher output efficiency. Reproduced with permission from ref 209. Copyright, Open access, MDPI publishing.

## 7 Next-Generation Nanogenerators for Healthcare Monitoring: Trends and Prospects

Nanogenerators are developing as powerful tools in the field of health monitoring because they can generate energy from natural sources such as body motion, heartbeats, and any vibrations. This unique ability makes it ideal for powering implants and wearable health sensors without the need for traditional batteries. As healthcare shifts toward more personalized and continuous monitoring, nanogenerators are expected to play a crucial role in making medical devices self-sufficient and reliable.

In the area of wearable health monitoring, nanogenerators will enable constant tracking of vital signs such as the heart rate, blood pressure, and breathing. Smart-fabric embedded with TENGs can harvest energy from body motion to power sensors that monitor muscle activity, hydration, or posture. For example, smart clothes worn by an athlete could track their performance in real time. Similarly, soft and flexible e-skin patches will serve as noninvasive biosensors that detect important biomarkers like glucose, sweat, and lactate. Devices such as wristbands or smart shoes with built-in nanogenerators can collect energy from walking or arm movement and use it for functions such as activity tracking, fall detection in elderly patients, or chronic disease management. NGs are also well-suited for implantable medical devices due to their compact size, safety, and ability to generate power from inside the body. Piezoelectric nanogenerators (PENGs), for example, can harvest energy from the heartbeat and power life-saving devices, such as pacemakers, reducing the need for battery replacement surgeries. In neuroscience, TENGs can be used in brain investigations to monitor brain signals and even deliver stimulation to treat conditions like epilepsy or Parkinson’s disease. Additionally, biodegradable NGs made from materials such as magnesium or PLGA can act as momentary monitoring systems after surgeries—like tracking brain pressure—and then safely dissolve in the body when their job is done.

The integration of artificial intelligence with self-sufficient systems will further improve health monitoring. Small AI models, such as TinyML, can analyze collected data in real time to detect health problems such as irregular heartbeat or low blood sugar levels without needing Internet or cloud support. Through advanced methods, such as federated learning, many nanogenerators can work together to create smart systems that learn from data without compromising patient privacy. Over time, these continuous data streams will help develop digital twin models of patients, allowing doctors to simulate disease progression and customize treatments accordingly. The advancement of nanogenerators will be heavily dependent on progress in materials science and structural design. New materials such as graphene-PVDF composites and zinc oxide nanowire arrays will help increase the energy output of nanogenerators. Self-healing materials that can heal themselves after damage will ensure long-term durability in the human body. In addition, using biodegradable and eco-friendly materials like silk or cellulose will help decrease electronic waste, especially in nonreusable medical applications.

One of the challenges of nanogenerators is that they do not always produce a steady supply of energy. To overcome this, future systems will combine multiple types of energy harvesting—such as combining TENGs and PENGs with solar or thermal elements. For instance, a wearable device could collect energy from both arm movements and body heat to work continuously. These hybrid systems will also include built-in energy storage solutions like micro supercapacitors or solid-route batteries that store extra power and provide bursts of energy when needed, such as for sending emergency alerts. To bring nanogenerators into everyday healthcare, they need to be mass-produced on a large scale and at a low cost. Techniques such as roll-to-roll printing can help produce flexible NGs quickly using affordable materials such as carbon nanotubes or conductive polymers. The use of 4D printing could also allow the development of sensors that change shape according to the body’s needs. As health data are collected and processed by these devices, ethical frameworks for AI will be necessary to ensure privacy, security, and fairness in the analysis. Nanogenerators can also make healthcare more accessible in underdeveloped or remote areas. For example, low-cost devices powered by a combination of solar and TENG technologies could detect diseases such as malaria in rural settings by analyzing changes in blood properties. For elderly patients, wearable nanogenerators could monitor risks, such as falls or cognitive decline, and automatically alert caregivers in emergencies, improving their safety and independence. However, to move these technologies from research laboratories to hospitals, many steps must still be taken. Regulatory approvals and clinical trials are needed to ensure safety and effectiveness. Tests must confirm that nanogenerator materials do not cause harmful immune responses. Close collaboration with agencies such as the FDA will help speed up support processes for these new self-sufficient medical devices. Also, large-scale testing across various environmental conditions, such as temperature and sweating, will be important to prove the real-world performance.

While nanogenerators hold enormous potential, certain challenges remain. Current power output levels (typically 1–100 μW/cm^2^) need to be increased to support more complex medical devices, such as artificial organs. Long-term reliability inside the body also needs to be proven. Additionally, concerns about data privacy and bias in AI algorithms must be addressed to ensure ethical use. Despite these challenges, the combination of nanogenerators with smart materials, AI, and sustainable manufacturing is set to transform healthcare by enabling preventive, personalized, and more accessible medical care. From heart patches that power themselves to AI-supported wearables, nanogenerators could help shift medicine from treating illnesses after they occur to preventing them before they start—improving health outcomes worldwide. This vision will only become reality through strong collaboration between researchers, doctors, and policy-makers.

## 8 Conclusions

The ability to harvest biomechanical energy from human motion and physiological activities has opened new possibilities for sustainable, self-powered biomedical systems. Nanogenerators (NGs), including piezoelectric, triboelectric, and hybrid types, efficiently convert low-frequency biomechanical stimuli into electrical energy, enabling the development of self-powered biomedical devices that function without external batteries. This advancement provides a foundation for autonomous, long-lasting, and maintenance-free healthcare devices. Integrating NGs into wearable and implantable platforms has significantly expanded their biomedical applications. Flexible NGs embedded in e-skins and patches facilitate continuous monitoring of motion, respiration, and physiological signals while simultaneously harvesting energy from daily activities. Implantable NGs within cardiovascular scaffolds, bone repair systems, or bladder-actuation devices deliver localized electrical cues for stimulation, regeneration, or actuation. Additionally, NG-based drug-release systems and biosensing patches enable controlled therapy and real-time diagnostics, while acoustic and ocular nanogenerators offer compact, self-sustained solutions for hearing restoration and eye-motion monitoring. NG-based technologies combine mechanical flexibility, biocompatibility, and high energy conversion efficiency, making them ideal for continuous, noninvasive healthcare applications. Future progress in material innovation, microfabrication, and integration with wireless communication and artificial intelligence (AI) will enhance the performance, adaptability, and intelligent data interpretation. Overall, nanogenerator-enabled self-powered systems represent a paradigm shift toward energy-autonomous, intelligent, and patient-centric healthcare, uniting energy harvesting, sensing, and therapeutic capabilities into a single sustainable platform for next-generation biomedical and wearable technologies.

## Data Availability

All data supporting the findings of this study are contained within the main manuscript. No additional data are available.

## References

[1] Zhang R., Hummelgård M., Örtegren J., Olsen M., Andersson H., Yang Y., Zheng H., Olin H. (2021). The triboelectricity
of the human body. Nano Energy.

[2] Dong K., Peng X., Wang Z. L. (2020). Fiber/fabric-based
piezoelectric
and triboelectric nanogenerators for flexible/stretchable and wearable
electronics and artificial intelligence. Adv.
Mater..

[3] Wang Z. L., Zhu G., Yang Y., Wang S., Pan C. (2012). Progress in Nanogenerators
for Portable Electronics. Mater. Today.

[4] Rocha-Ortiz G., Gonzalez-Gutierrez A. G., Casillas-Santana N., Astudillo-Sanchez P.
D., Romero-Arellano V. H., Blancas-Flores J. M. (2025). Innovative
dielectric polyaniline composites for performance triboelectric nanogenerators. J. Sol. Stat Electroch..

[5] Islam E., Abdullah A. M., Chowdhury A. R., Tasnim F., Martinez M., Olivares C., Lozano K., Uddin M. J. (2020). Electromagnetic-triboelectric-hybrid
energy tile for biomechanical green energy harvesting. Nano Energy.

[6] Karan S. K., Maiti S., Lee J. H., Mishra Y. K., Khatua B. B., Kim J. K. (2020). Recent Advances
in Self-Powered Tribo-/Piezoelectric
Energy Harvesters: All-In-One Package for Future Smart Technologies. Adv. Funct. Mater..

[7] Pan S., Zhang Z. (2017). Triboelectric Effect:
A New Perspective on Electron Transfer Process. J. Appl. Phys..

[8] Zi Y., Basset P., Chen J. (2021). Triboelectric
nanogenerator—Progress
and perspectives. EcoMat.

[9] Wen H., Yang P., Liu G., Xu S., Yao H., Li W., Qu H., Ding J., Li J., Wan L. (2022). Flower-like
triboelectric nanogenerator for blue energy harvesting with six degrees
of freedom. Nano Energy.

[10] Kumar B., Kim S. W. (2011). Recent Advances in Power Generation through Piezoelectric
Nanogenerators. J. Mater. Chem..

[11] Zhao Z., Wang J. (2025). Advances in Interfacial
Electrostatic Energy Harvesting via Direct
Current Triboelectric Nanogenerators. Adv. En.
Mater..

[12] Fan F.-R., Tian Z. Q., Lin Wang Z. (2012). Flexible triboelectric
generator. Nano Energy.

[13] Shi B., Liu Z., Zheng Q., Meng J., Ouyang H., Zou Y., Jiang D., Qu X., Yu M., Zhao L. (2019). Body-integrated self-powered
system for wearable and implantable
applications. ACS Nano.

[14] Wang Y., Hong M., Venezuela J., Liu T., Dargusch M. (2023). Expedient
secondary functions of flexible piezoelectrics for biomedical energy
harvesting. Bioa. Mater..

[15] Lu B., Jia S., Wang Z., Wu W., Yan L., Zhu L., Hao D. (2023). Sensory-motor coupling
electrical stimulation driven by a bionic
Z-structured triboelectric nanogenerator improves functional recovery
from spinal cord injury. Nano Energy.

[16] Cheedarala R. K., Song J. I. (2022). Integrated electronic skin (e-skin)
for harvesting
of TENG energy through push–pull ionic electrets and ion-ion
hopping mechanism. Sci. Rep..

[17] Wang X., Gu Y., Xiong Z., Cui Z., Zhang T. (2014). Silk-molded flexible,
ultrasensitive, and highly stable electronic skin for monitoring human
physiological signals. Adv. Mater..

[18] Xu S., Qin Y., Xu C., Wei Y., Yang R., Wang Z. L. (2010). Self-powered
nanowire devices. Nat. Nanotechnol..

[19] Cheng R., Ning C., Chen P., Sheng F., Wei C., Zhang Y., Peng X., Dong K., Wang Z. L. (2022). Enhanced
Output of On-Body Direct-Current Power Textiles by Efficient Energy
Management for Sustainable Working of Mobile Electronics. Adv. Ener. Mater..

[20] Chen J., Ren Y., Xiang H., Jiang X., Yang X., Guo H. (2022). A self-powered
human-pet interaction system enabled by triboelectric nanogenerator
functionalized pet-leash. Nano Energy.

[21] Gui Y., Wang Y., He S., Yang J. (2022). Self-powered smart
agriculture real-time sensing device based on hybrid wind energy harvesting
triboelectric-electromagnetic nanogenerator. Energy Convers. Manage..

[22] Dassanayaka D. G., Alves T. M., Wanasekara N. D., Dharmasena I. G., Ventura J. (2022). Recent progresses in wearable triboelectric
nanogenerators. Adv. Func. Mater..

[23] Shen S., Xiao X., Yin J., Xiao X., Chen J. (2022). Self-powered
smart gloves based on triboelectric nanogenerators. Small Methods.

[24] Ma J., Youn J. H., Cho H., Park J., Kyung K. U. (2022). Highly
efficient long-lasting triboelectric nanogenerator upon impact and
its application to daily-life self-cleaning solar panel. Nano Energy.

[25] Wang X., Shi Y., Yang P., Tao X., Li S., Lei R., Liu Z., Wang Z. L., Chen X. (2022). Fish-wearable data snooping platform
for underwater energy harvesting and fish behavior monitoring. Small.

[26] Lee C., Park H., Lee J. H. (2020). Recent
structure development of poly
(vinylidene fluoride)-based piezoelectric nanogenerator for self-powered
sensor. Actuators.

[27] Mokhtari F., Danti S., Azimi B., Hellies F., Zanoletti E., Albertin G., Astolfi L., Varley R. J., Razal J. M. (2025). Self-Powered
Nanostructured Piezoelectric Filaments as Advanced Transducers for
New Cochlear Implants. EEM.

[28] Vera
Anaya D., He T., Lee C., Yuce M. R. (2020). Self-powered
eye motion sensor based on triboelectric interaction and near-field
electrostatic induction for wearable assistive technologies. Nano Energy.

[29] Wang Z. L. (2022). On the
expanded Maxwell’s equations for moving charged media system–General
theory, mathematical solutions and applications in TENG. Mater. Today.

[30] Liu Y., Wang L., Zhao L., Yu X., Zi Y. (2020). Recent progress
on flexible nanogenerators toward self-powered systems. InfoMat.

[31] Das N., Sarkar D., Saikh M. M., Biswas P., Das S., Hoque N. A., Ray P. P. (2022). Piezoelectric activity assessment
of size-dependent naturally acquired mud volcano clay nanoparticles
assisted highly pressure sensitive nanogenerator for green mechanical
energy harvesting and body motion sensing. Nano
Energy.

[32] Sarkar D., Das N., Saikh M. M., Biswas P., Roy S., Paul S., Hoque N. A., Basu R., Das S. (2022). High β-crystallinity
comprising nitrogenous carbon dot/PVDF nanocomposite decorated self-powered
and flexible piezoelectric nanogenerator for harvesting human movement
mediated energy and sensing weights. Ceram.
Int..

[33] Uchino, K. Fundamentals of Piezoelectrics. In Encyclopedia of Smart Materials, Elsevier, 2022; pp 1–21.

[34] Mahapatra S. D., Mohapatra P. C., Aria A. I., Christie G., Mishra Y. K., Hofmann S., Thakur V. K. (2021). Piezoelectric materials for energy
harvesting and sensing applications: Roadmap for future smart materials. Adv. Sci..

[35] Tichý, J. ; Erhart, J. ; Kittinger, E. ; Prívratská, J. Fundamentals of piezoelectric sensorics: mechanical, dielectric, and thermodynamical properties of piezoelectric materials; Springer Science & Business Media, 2010.

[36] Xu Q., Wen J., Qin Y. (2021). Development and Outlook of High-Output
Piezoelectric
Nanogenerators. Nano Energy.

[37] Wang Y., Guo X., Li L. H., Zhang J., Li G. K., Zavabeti A., Li Y. (2022). Enhanced piezoelectric properties enabled by engineered low-dimensional
nanomaterials. ACS Appl. Nano Mater..

[38] Pandey R. K., Dutta J., Brahma S., Rao B., Liu C. P. (2021). Review
on ZnO-based piezotronics and piezoelectric nanogenerators: aspects
of piezopotential and screening effect. J. Phys.
Mater.

[39] Yan X., Li G., Wang Z., Yu Z., Wang K., Wu Y. (2020). Recent progress
on piezoelectric materials for renewable energy conversion. Nano Energy.

[40] Sripadmanabhan
Indira S., Aravind Vaithilingam C., Oruganti K. S. P., Mohd F., Rahman S. (2019). Nanogenerators as a sustainable power
source: state of art, applications, and challenges. Nanomaterials.

[41] Liu H., Zhong J., Lee C., Lee S. W., Lin L. (2018). A comprehensive
review on piezoelectric energy harvesting technology: Materials, mechanisms,
and applications. Appl. Phys. Rev..

[42] Li Y., Liu X., Ren Z., Luo J., Zhang C., Cao C. C., Yuan H., Pang Y. (2024). Marine biomaterial-based triboelectric
nanogenerators: insights and applications. Nano
Energy.

[43] Panda S., Hajra S., Oh Y., Oh W., Lee J., Shin H., Vivekananthan V., Yang Y., Mishra Y. K., Kim H. J. (2023). Hybrid nanogenerators
for ocean energy harvesting:
mechanisms, designs, and applications. Small.

[44] Zi Y. L., Lin L., Wang J., Wang S. H., Chen J., Fan X., Yang P.-K., Yi F., Wang Z. L. (2015). Triboelectric–pyroelectric–
piezoelectric hybrid cell for high-efficiency energy-harvesting and
self-powered sensing. Adv. Mater..

[45] Jung W.-S., Kang M.-G., Moon H. G., Baek S.-H., Yoon S.-J., Wang Z. L., Kim S.-W., Kang C.-Y. (2015). High output
piezo/triboelectric
hybrid generator. Sci. Rep..

[46] Bouhamed A., Missaoui S., Ben Ayed A., Attaoui A., Missaoui D., Jeder K., Guesmi N., Njeh A., Khemakhem H., Kanoun O. (2024). A comprehensive review
of strategies toward efficient
flexible piezoelectric polymer composites based on BaTiO_3_ for next-generation energy harvesting. Energies.

[47] Nishiyama T., Sumihara T., Sato E., Horibe H. (2017). Effect of solvents
on the crystal formation of poly (vinylidene fluoride) film prepared
by a spin-coating process. Polym. J..

[48] Divya S., Hemalatha J. (2017). Study on the
enhancement of ferroelectric β phase
in P (VDF-HFP) films under heating and poling conditions. Eur. Polym. J..

[49] Ramasundaram S., Yoon S., Kim K. J., Lee J. S. (2008). Direct preparation
of nanoscale thin films of poly (vinylidene fluoride) containing β-crystalline
phase by heat-controlled spin coating. Macromol.
Chem. Phys..

[50] Cardoso V. F., Costa C. M., Minas G., Lanceros-Mendez S. (2012). Improving
the optical and electroactive response of poly (vinylidene fluoride–trifluoroethylene)
spin-coated films for sensor and actuator applications. Smart Mater. Struct..

[51] Barhoum A., Pal K., Rahier H., Uludag H., Kim I. S., Bechelany M. (2019). Nanofibers
as new-generation materials: From spinning and nano-spinning fabrication
techniques to emerging applications. Appl. Mater.
Today.

[52] Ibrahim Y. S., Hussein E. A., Zagho M. M., Abdo G. G., Elzatahry A. A. (2019). Melt electrospinning
designs for nanofiber fabrication for different applications. Int. J. Mol. Sci..

[53] Dadol G. C., Kilic A., Tijing L. D., Lim K. J. A., Cabatingan L. K., Tan N. P. B., Stojanovska E., Polat Y. (2020). Solution blow spinning
(SBS) and SBS-spun nanofibers: Materials, methods, and applications. Mater. Today Commun..

[54] Cheon S., Kang H., Kim H., Son Y., Lee J. Y., Shin H. J., Kim S., Cho J. H. (2018). High-performance
triboelectric nanogenerators based on electrospun polyvinylidene fluoride–silver
nanowire composite nanofibers. Adv. Funct. Mater..

[55] Kriegel C., Arrechi A., Kit K., McClements D. J., Weiss J. (2008). Fabrication, functionalization, and application of electrospun biopolymer
nanofibers. Crit. Rev. Food Sci. Nutr..

[56] Cho Y., Beak J. W., Sagong M., Ahn S., Nam J. S., Kim I. D. (2025). Electrospinning and nanofiber technology:
fundamentals,
innovations, and applications. Adv. Mater..

[57] Cai J., Niu H., Li Z., Du Y., Cizek P., Xie Z., Xiong H., Lin T. (2015). High-performance supercapacitor electrode
materials from cellulose-derived carbon nanofibers. ACS Appl. Mater. Interfaces.

[58] Yang Y., Guo W., Pradel K. C., Zhu G., Zhou Y., Zhang Y., Hu Y., Lin L., Wang Z. L. (2012). Pyroelectric nanogenerators for harvesting
thermoelectric energy. Nano Lett..

[59] Paranjape M. V., Graham S. A., Manchi P., Kurakula A., Yu J. S. (2023). Multistage
SrBaTiO_3_/PDMS Composite Film-Based Hybrid Nanogenerator
for Efficient Floor Energy Harvesting Applications. Small.

[60] Ponnamma D., Aljarod O., Parangusan H., Ali Al-Maadeed M. A. (2020). Electrospun
nanofibers of PVDF-HFP composites containing magnetic nickel ferrite
for energy harvesting application. Mater. Chem.
Phys..

[61] Amrutha B., Prasad G., Sathiyanathan P., Reza M. S., Kim H., Pathak M., Prabu A. A. (2023). Fabrication
of CuO-NP-doped PVDF
composites based electrospun triboelectric nanogenerators for wearable
and biomedical applications. Polymers.

[62] Sengupta P., Ghosh A., Bose N., Mukherjee S., Roy Chowdhury A., Datta P. (2020). A comparative assessment of poly
(vinylidene fluoride)/conducting polymer electrospun nanofiber membranes
for biomedical applications. J. Appl. Polym.
Sci..

[63] Yang J., Zhang Y., Li Y., Wang Z., Wang W., An Q., Tong W. (2021). Piezoelectric
nanogenerators based on graphene oxide/PVDF
electrospun nanofiber with enhanced performances by in-situ reduction. Mater. Today Commun..

[64] Chinya I., Sasmal A., Pal A., Sen S. (2019). Flexible piezoelectric
energy harvesters using different architectures of ferrite-based nanocomposites. CrystEngComm.

[65] Sasmal A., Medda S. K., Devi P. S., Sen S. (2020). Nano-ZnO decorated
ZnSnO_3_ as efficient fillers in PVDF matrixes: Toward simultaneous
enhancement of energy storage density and efficiency and improved
energy harvesting activity. Nanoscale.

[66] Xi B., Wang L., Yang B., Xia Y., Chen D., Wang X. (2023). Boosting output performance of triboelectric
nanogenerator based
on BaTiO_3_: La embedded nanofiber membrane for energy harvesting
and wireless power transmission. Nano Energy.

[67] Bi M., Hao Y., Zhang J., Lei M., Bi K. (2017). Particle size
effect
of BaTiO_3_ nanofillers on the energy storage performance
of polymer nanocomposites. Nanoscale.

[68] An S., Jo H. S., Li G., Samuel E., Yoon S. S., Yarin A. L. (2020). Sustainable nanotextured
wave energy harvester based
on ferroelectric fatigue-free and flexoelectricity-enhanced piezoelectric
P (VDF-TrFE) nanofibers with BaSrTiO_3_ nanoparticles. Adv. Funct. Mater..

[69] Patra A., Pal A., Sen S. (2018). Polyvinylpyrrolidone modified barium zirconate titanate/polyvinylidene
fluoride nanocomposites as self-powered sensor. Ceram. Int..

[70] Rastegardoost M. M., Tafreshi O. A., Saadatnia Z., Ghaffari-Mosanenzadeh S., Park C. B., Naguib H. E. (2023). Porous PVDF mats with significantly
enhanced dielectric properties and novel dipole arrangement for high-performance
triboelectric nanogenerators. Appl. Mater. Today.

[71] Singh V., Singh B. (2023). MoS2-PVDF/PDMS based flexible hybrid
piezo-triboelectric nanogenerator
for harvesting mechanical energy. J. Alloys
Compd..

[72] Kar E., Bose N., Dutta B., Banerjee S., Mukherjee N., Mukherjee S. (2019). 2D SnO_2_ Nanosheet/PVDF Composite-Based Flexible,
Self-Cleaning Piezoelectric Energy Harvester. Energy Convers. Manag..

[73] Muduli S. P., Veeralingam S., Badhulika S. (2022). Multilayered Piezoelectric Nanogenerator
Based on Lead-Free Poly­(vinylidene fluoride)-(0.67 BiFeO_3_-0.33 BaTiO_3_) Electrospun Nanofiber Mats for Fast Charging
of Supercapacitors. ACS Appl. Energy Mater..

[74] Islam M. J., Lee H., Lee K., Cho C., Kim B. (2023). Piezoelectric Nanogenerators
Fabricated Using Spin Coating of Poly­(vinylidene fluoride) and ZnO
Composite. Sci. Rep..

[75] Serairi L., Leprince-Wang Y. (2022). ZnO nanowire-based
piezoelectric nanogenerator device
performance tests. Crystals.

[76] Wang Z. L., Song J. (2006). Piezoelectric nanogenerators based on zinc oxide nanowire arrays. Science.

[77] Yan J., Jeong Y. G. (2016). High performance flexible piezoelectric nanogenerators
based on BaTiO_3_ nanofibers in different alignment modes. ACS Appl. Mater. Interfaces.

[78] Khadtare S., Kumar A., Jung U., Sang C., Park J. (2022). Piezoelectric
nanogenerator based on lead-free BiFeO_3_: Sr perovskite. J. Alloys Compd..

[79] Abdullah A. M., Sadaf M. U. K., Tasnim F., Vasquez H., Lozano K., Uddin M. J. (2021). KNN based piezo-triboelectric
lead-free hybrid energy
films. Nano Energy.

[80] Park K.-I., Son J. H., Hwang G. T., Jeong C. K., Ryu J., Koo M., Choi I., Lee S. H., Byun M., Wang Z. L. (2014). Highly-efficient, flexible piezoelectric PZT thin film nanogenerator
on plastic substrates. Adv. Mater..

[81] Rovisco A., Dos Santos A., Cramer T., Martins J., Branquinho R., Águas H., Fraboni B., Fortunato E., Martins R., Igreja R. (2020). Piezoelectricity enhancement
of nanogenerators based on PDMS and ZnSnO_3_ nanowires through
microstructuration. ACS Appl. Mater. Interfaces.

[82] Tao H., Yu X., Li L., Angeles M. D. L., Wang N. (2025). Freeze-resistant wearable
strain sensors based on hyperbranched cellulose nanofiber hydrogels. Results Eng..

[83] Chen Y., Shi C., Zhang J., Dai Y., Su Y., Liao B., Zhang M., Tao X., Zeng W. (2022). Ionic thermoelectric
effect inducing cation-enriched surface of hydrogel to enhance output
performance of triboelectric nanogenerator. Energy Technol..

[84] Guo R., Fang Y., Wang Z., Libanori A., Xiao X., Wan D., Cui X., Sang S., Zhang W., Zhang H., Chen J. (2022). Deep learning
assisted body area triboelectric hydrogel sensor network
for infant care. Adv. Funct. Mater..

[85] He H., Liu J., Wang Y., Zhao Y., Qin Y., Zhu Z., Yu Z., Wang J. (2022). An ultralight self-powered fire alarm
e-textile based
on conductive aerogel fiber with repeatable temperature monitoring
performance used in firefighting clothing. ACS
Nano.

[86] Hu K., Zhao Z., Wang Y., Yu L., Liu K., Wu H., Huang L., Chen L., Ni Y. (2022). A tough organohydrogel-based
multiresponsive sensor for a triboelectric nanogenerator and supercapacitor
toward wearable intelligent devices. J. Mater.
Chem. A.

[87] Jing X., Li H., Mi H. Y., Feng P. Y., Tao X., Liu Y., Liu C., Shen C. (2020). Enhancing the performance of a stretchable and transparent
triboelectric nanogenerator by optimizing the hydrogel ionic electrode
property. ACS Appl. Mater. Interfaces.

[88] Liang Q., Zhang Q., Yan X., Liao X., Han L., Yi F., Ma M., Zhang Y. (2017). Recyclable and green triboelectric
nanogenerator. Adv. Mater..

[89] Liu T., Liu M., Dou S., Sun J., Cong Z., Jiang C., Du C., Pu X., Hu W., Wang Z. L. (2018). Triboelectric-nanogenerator-based
soft energy-harvesting skin enabled by toughly bonded elastomer/hydrogel
hybrids. ACS Nano.

[90] Pang Y., Xi F., Luo J., Liu G., Guo T., Zhang C. (2018). An alginate
film-based degradable triboelectric nanogenerator. RSC Adv..

[91] Saqib Q. M., Chougale M. Y., Khan M. U., Shaukat R. A., Kim J., Bae J., Lee H. W., Park J. I., Kim M. S., Lee B. G. (2021). Natural
seagrass tribopositive material based spray coatable triboelectric
nanogenerator. Nano Energy.

[92] Xia K., Wu D., Fu J., Hoque N. A., Ye Y., Xu Z. (2020). Tunable output
performance of triboelectric nanogenerator based on alginate metal
complex for sustainable operation of intelligent keyboard sensing
system. Nano Energy.

[93] Charoonsuk T., Pongampai S., Pakawanit P., Vittayakorn N. (2021). Achieving
a highly efficient chitosan-based triboelectric nanogenerator via
adding organic proteins: Influence of morphology and molecular structure. Nano Energy.

[94] Charoonsuk T., Supansomboon S., Pakawanit P., Vittayakorn W., Pongampai S., Woramongkolchai S., Vittayakorn N. (2022). Simple enhanced
charge density of chitosan film by the embedded ion method for the
flexible triboelectric nanogenerator. Carbohydr.
Polym..

[95] Cheedarala R. K., Song J. I. (2021). Moderately transparent
chitosan-PVA blended membrane
for strong mechanical stiffness and as a robust bio-material energy
harvester through contact-separation mode TENG. Front. Nanotechnol..

[96] Huang J., Hao Y., Zhao M., Qiao H., Huang F., Li D., Wei Q. (2021). Biomass-based
wearable and Self-powered pressure sensor for human
motion detection. Composites, Part A.

[97] Tao K., Chen Z., Yu J., Zeng H., Wu J., Wu Z., Jia Q., Li P., Fu Y., Chang H., Yuan W. (2022). Ultra-sensitive, deformable,
and transparent triboelectric tactile
sensor based on micro-pyramid patterned ionic hydrogel for interactive
human–machine interfaces. Adv. Sci..

[98] Liu B., Wang S., Yuan Z., Duan Z., Zhao Q., Zhang Y., Su Y., Jiang Y., Xie G., Tai H. (2020). Novel chitosan/ZnO bilayer film with enhanced humidity-tolerant property:
Endowing triboelectric nanogenerator with acetone analysis capability. Nano Energy.

[99] Liu B. H., Xie G. Z., Li C. Z., Wang S., Yuan Z., Duan Z. H., Jiang Y. D., Tai H. L. (2021). A chitosan/amido-graphene
oxide-based self-powered humidity sensor enabled by the triboelectric
effect. Rare Metals.

[100] Shen S., Yi J., Sun Z., Guo Z., He T., Ma L., Li H., Fu J., Lee C., Wang Z. L. (2022). Human machine interface with wearable electronics using
biodegradable triboelectric films for calligraphy practice and correction. Nano-Micro Lett..

[101] Somseemee O., Sae-Oui P., Siriwong C. (2022). Bio-based
epoxidized
natural rubber/chitosan/cellulose nanocrystal composites for enhancing
mechanical properties, self-healing behavior and triboelectric nanogenerator
performance. Cellulose.

[102] Weldemhret T. G., Lee D. W., Prabhakar M. N., Park Y. T., Song J. I. (2022). Polyurethane foams coated with phosphorus-doped
mesoporous carbon for flame-retardant triboelectric nanogenerators. ACS Appl. Nano Mater..

[103] Xiang H., Yang J., Cao X., Wang N. (2022). Flexible and
highly sensitive triboelectric nanogenerator with magnetic nanocomposites
for cultural heritage conservation and human motion monitoring. Nano Energy.

[104] Yan K., Li X., Wang X. X., Yu M., Fan Z., Ramakrishna S., Hu H., Long Y. Z. (2020). A non-toxic
triboelectric
nanogenerator for baby care applications. J.
Mater. Chem. A.

[105] Zheng Z., Yu D., Wang B., Guo Y. (2022). Ultrahigh
sensitive, eco-friendly, transparent triboelectric nanogenerator for
monitoring human motion and vehicle movement. Chem. Eng. J..

[106] Chiu C. M., Chen S. W., Pao Y. P., Huang M. Z., Chan S. W., Lin Z. H. (2019). A smart glove with
integrated triboelectric
nanogenerator for self-powered gesture recognition and language expression. Sci. Technol. Adv. Mater..

[107] Eom K., Shin Y. E., Kim J. K., Joo S. H., Kim K., Kwak S. K., Ko H., Jin J., Kang S. J. (2020). Tailored
poly (vinylidene fluoride-co-trifluoroethylene) crystal orientation
for a triboelectric nanogenerator through epitaxial growth on a chitin
nanofiber film. Nano Lett..

[108] Sun J., Choi H., Cha S., Ahn D., Choi M., Park S., Cho Y., Lee J., Park T. E., Park J. J. (2022). Highly enhanced triboelectric performance
from increased
dielectric constant induced by ionic and interfacial polarization
for chitosan based multi-modal sensing system. Adv. Funct. Mater..

[109] Lu D., Liu T., Meng X., Luo B., Yuan J., Liu Y., Zhang S., Cai C., Gao C., Wang J., Wang S. (2023). Wearable triboelectric
visual sensors for tactile perception. Adv.
Mater..

[110] Jao Y.-T., Yang P. K., Chiu C. M., Lin Y. J., Chen S. W., Choi D., Lin Z. H. (2018). A textile-based
triboelectric nanogenerator with humidity-resistant output characteristics
and its applications in self-powered healthcare sensors. Nano Energy.

[111] Kim J. N., Lee J., Go T. W., Rajabi-Abhari A., Mahato M., Park J. Y., Lee H., Oh I. K. (2020). Skin-attachable
and biofriendly chitosan-diatom triboelectric nanogenerator. Nano Energy.

[112] Kim J. N., Lee J., Lee H., Oh I. K. (2021). Stretchable
and self-healable catechol-chitosan-diatom hydrogel for triboelectric
generator and self-powered tremor sensor targeting at Parkinson disease. Nano Energy.

[113] Ma C., Gao S., Gao X., Wu M., Wang R., Wang Y., Tang Z., Fan F., Wu W., Wan H., Wu W. (2019). Chitosan biopolymer-derived self-powered
triboelectric
sensor with optimized performance through molecular surface engineering
and data-driven learning. InfoMat.

[114] Peng X., Dong K., Zhang Y., Wang L., Wei C., Lv T., Wang Z. L., Wu Z. (2022). Sweat-permeable, biodegradable,
transparent and self-powered chitosan-based electronic skin with ultrathin
elastic gold nanofibers. Adv. Funct. Mater..

[115] Pongampai S., Charoonsuk T., Pinpru N., Pulphol P., Vittayakorn W., Pakawanit P., Vittayakorn N. (2021). Triboelectric-piezoelectric
hybrid nanogenerator based on BaTiO_3_-Nanorods/Chitosan
enhanced output performance with self-charge-pumping system. Composites, Part B.

[116] El-Ghoul Y., Alminderej F. M., Alsubaie F. M., Alrasheed R., Almousa N. H. (2021). Recent advances
in functional polymer materials for
energy, water, and biomedical applications: a review. Polymers.

[117] Wang L., Daoud W. A. (2019). Hybrid conductive
hydrogels for a
washable human motion energy harvester and a self-powered temperature-stress
dual sensor. Nano Energy.

[118] Wang R., Gao S., Yang Z., Li Y., Chen W., Wu B., Wu W. (2018). Engineered and laser-processed
chitosan biopolymers for sustainable and biodegradable triboelectric
power generation. Adv. Mater..

[119] Zhang J., Hu Y., Lin X., Qian X., Zhang L., Zhou J., Lu A. (2022). High-performance triboelectric
nanogenerator based on chitin for mechanical-energy harvesting and
self-powered sensing. Carbohydr. Polym..

[120] Han X., Jiang D., Qu X., Bai Y., Cao Y., Luo R., Li Z. (2021). A stretchable, self-healable triboelectric
nanogenerator
as electronic skin for energy harvesting and tactile sensing. Materials.

[121] Ma J., Zhu J., Ma P., Jie Y., Wang Z. L., Cao X. (2020). Fish bladder film-based triboelectric
nanogenerator for noncontact
position monitoring. ACS Energy Lett..

[122] Han Y., Han Y., Zhang X., Li L., Zhang C., Liu J., Lu G., Yu H. D., Huang W. (2020). Fish gelatin based
triboelectric nanogenerator for harvesting biomechanical energy and
self-powered sensing of human physiological signals. ACS Appl. Mater. Interfaces.

[123] Sun Q., Wang L., Yue X., Zhang L., Ren G., Li D., Wang H., Han Y., Xiao L., Lu G., Yu H. D. (2021). Fully sustainable and high-performance fish
gelatin-based
triboelectric nanogenerator for wearable movement sensing and human-machine
interaction. Nano Energy.

[124] Zhang D., Yang Y., Xu Z., Wang D., Du C. (2022). An eco-friendly
gelatin based triboelectric nanogenerator for a self-powered
PANI nanorod/NiCo_2_O_4_ nanosphere ammonia gas
sensor. J. Mater. Chem. A.

[125] Chen Z., Yu J., Zeng H., Chen Z., Tao K., Wu J., Li Y. (2021). An electret/hydrogel-based tactile
sensor boosted by micro-patterned and electrostatic promoting methods
with flexibility and wide-temperature tolerance. Micromachines.

[126] Li X., Xiang S., Ling D., Zhang S., Li C., Dai R., Zhu P., Liu X., Pan Z. (2022). Stretchable, self-healing,
transparent macromolecular elastomeric gel and PAM/carrageenan hydrogel
for self-powered touch sensors. Mater. Sci.
Eng.: B.

[127] Kang M., Bin Mohammed Khusrin M. S., Kim Y. J., Kim B., Park B. J., Hyun I., Imani I. M., Choi B. O., Kim S. W. (2022). Nature-derived highly
tribopositive ϰ-carrageenan-agar
composite-based fully biodegradable triboelectric nanogenerators. Nano Energy.

[128] Tao K., Chen Z., Yu J., Zeng H., Wu J., Wu Z., Jia Q., Li P., Fu Y., Chang H., Yuan W. (2022). Ultra-sensitive, deformable,
and transparent triboelectric tactile
sensor based on micro-pyramid patterned ionic hydrogel for interactive
human–machine interfaces. Adv. Sci..

[129] Liu W., Shi Y., Sun Z., Zhang L. (2022). Poling-free
hydroxyapatite/polylactide
nanogenerator with improved piezoelectricity for energy harvesting. Micromachines.

[130] Khandelwal G., Min G., Karagiorgis X., Dahiya R. (2023). Aligned PLLA electrospun fibres based biodegradable
triboelectric nanogenerator. Nano Energy.

[131] Mi Y., Lu Y., Shi Y., Zhao Z., Wang X., Meng J., Cao X., Wang N. (2023). Biodegradable
Polymers
in Triboelectric Nanogenerators. Polymers.

[132] Hu S., Han J., Shi Z., Chen K., Xu N., Wang Y., Zheng R., Tao Y., Sun Q., Wang Z. L., Yang G. (2022). Biodegradable, super-strong, and
conductive cellulose macrofibers for fabric-based triboelectric nanogenerator. Nano-Micro Lett..

[133] Kim S., Gupta M. K., Lee K. Y., Sohn A., Kim T. Y., Shin K. S., Kim D., Kim S. K., Lee K. H., Shin H. J., Kim D. W. (2014). Transparent flexible
graphene triboelectric nanogenerators. Adv.
Mater.:Compos. Carbon, Pap. Symp..

[134] Oh J., Kang M., Park J., Kim T. Y., Park K., Seo J. (2024). Autonomously Self-healing, Adhesive, and Stretchable Triboelectric
Nanogenerator Using Multifunctional Hydrogel-Elastomer Double Layer
with a Power Management Circuit. Adv. Electron.
Mater..

[135] Liu Y., Li E., Yan Y., Lin Z., Chen Q., Wang X., Shan L., Chen H., Guo T. (2021). A one-structure-layer
PDMS/Mxenes based stretchable triboelectric nanogenerator for simultaneously
harvesting mechanical and light energy. Nano
Energy.

[136] Maria Joseph Raj N. P., Abisegapriyan K. S., Khandelwal G., Alluri N. R., Kim S. J. (2020). A lead-free ferroelectric
Bi_0.5_Na_0.5_TiO_3_ based flexible, lightweight
nanogenerator for motion monitoring applications. Sustainable Energy Fuels.

[137] Zhang Y., Li H., Wang E., Gao J., Yue L., Zhao M., Zhang L. (2024). Mn-Doped NaNbO_3_/Na_0.5_Bi_0.5_TiO_3_ Lead-Free Ferroelectric
Ceramics with Enhanced Energy Storage Performance. Coatings.

[138] Guan Q., Lin G., Gong Y., Wang J., Tan W., Bao D., Liu Y., You Z., Sun X., Wen Z., Pan Y. (2019). Highly efficient self-healable
and dual responsive
hydrogel-based deformable triboelectric nanogenerators for wearable
electronics. J. Mater. Chem. A.

[139] Wang L., Daoud W. A. (2019). High-performance
flexible piezoelectric
nanogenerator based on perovskite nanostructures for energy harvesting
applications. Nano Energy.

[140] Zhang B., Ren T., Li H., Chen B., Mao Y. (2024). Recent Progress of
Nature Materials–Based Triboelectric Nanogenerators
for Electronic Skins and Human–Machine Interaction. Adv. Energy Sustainability Res..

[141] Bao D., Wen Z., Shi J., Xie L., Jiang H., Jiang J., Yang Y., Liao W., Sun X. (2020). An anti-freezing
hydrogel-based stretchable triboelectric nanogenerator for biomechanical
energy harvesting at sub-zero temperature. J.
Mater. Chem. A.

[142] Yiming B., Han Y., Han Z., Zhang X., Li Y., Lian W., Zhang M., Yin J., Sun T., Wu Z., Li T., Fu J., Jia Z., Qu S. (2021). An ultra-stretchable
and self-healing triboelectric nanogenerator for wearable self-powered
sensors. Adv. Mater..

[143] Hu Y., Zhang M., Qin C., Qian X., Zhang L., Zhou J., Lu A. (2021). Biodegradable
and flexible cellulose-based
triboelectric nanogenerator for self-powered wearable devices. Carbohydr. Polym..

[144] Liu P., Sun N., Mi Y., Luo X., Dong X., Cai J., Jia X., Ramos M. A., Hu T. S., Xu Q. (2021). Ultra-Low
CNTs Filled High-Performance Fast Self-Healing Triboelectric Nanogenerators
for Wearable Electronics. Compos. Sci. Technol..

[145] Wu Y., Qu J., Zhang X., Ao K., Zhou Z., Zheng Z., Mu Y., Wu X., Luo Y., Feng S. P. (2021). Biomechanical Energy Harvesters Based on Ionic Conductive
Organohydrogels via the Hofmeister Effect and Electrostatic Interaction. ACS Nano.

[146] Wang Z. L. (2013). Triboelectric nanogenerators as new energy technology
for self-powered systems and as active mechanical and chemical sensors. ACS Nano.

[147] Dagdeviren C., Yang B. D., Su Y., Tran P. L., Joe P., Anderson E., Xia J., Doraiswamy V., Dehdashti B., Feng X., Lu B., Poston R., Khalpey Z., Ghaffari R., Huang Y., Slepian M. J., Rogers J. A. (2015). Conformal piezoelectric systems for
clinical and experimental
characterization of soft tissue biomechanics. Nat. Mater..

[148] Pu X., Li L., Song H., Du C., Zhao Z., Jiang C., Cao G., Hu W., Wang Z. L. (2016). Wearable
self-charging power textile based on flexible yarn supercapacitors
and triboelectric nanogenerators. Adv. Mater..

[149] You Z., Wang S., Li Z., Zou Y., Lu T., Wang F., Hu B., Wang X., Li L., Fang W., Liu Y. (2022). High Current Output Direct-Current
Triboelectric Nanogenerator Based on Organic Semiconductor Heterojunction. Nano Energy.

[150] El-Mohandes A. M., Zheng R. (2021). Active Matching Circuit
to Enhance
the Generated Power of Triboelectric Nanogenerators. Nano Energy.

[151] Trung T. Q., Lee N. E. (2016). Flexible and stretchable
physical
sensor integrated platforms for wearable human-activity monitoring
and personal healthcare. Adv. Mater..

[152] Javaid S., Fahim H., Zeadally S., He B. (2023). Self-Powered
Sensors: Applications, Challenges, and Solutions. IEEE Sens. J..

[153] Mao J., Zhou P., Wang X., Yao H., Liang L., Zhao Y., Zhang J., Ban D., Zheng H. (2023). A Health Monitoring
System Based on Flexible Triboelectric Sensors for Intelligent Medical
Internet of Things and Its Applications in Virtual Reality. Nano Energy.

[154] Dong L., Zhao C., Han C., Yang Y., Yang F. (2025). Advancement of AI-Assisted Self-Powered
Healthcare Sensing Systems. Med. Mat..

[155] Liu L., Guo X., Liu W., Lee C. (2021). Recent Progress in
the Energy Harvesting Technology—From Self-Powered Sensors
to Self-Sustained IoT, and New Applications. Nanomaterials.

[156] Raffik, R. ; Ali, L. F. ; Maria, W. A. ; Subashini, B. ; Asvitha, R. Integration of Internet of Medical Things (IoMT) and Artificial Intelligence Applications in Healthcare and Medicine: A Multidisciplinary Perspective. In Industry 5.0 for Society 5.0: Advancing Medical IoT and Smart Healthcare (Part 1); Bentham Science Publishers: 2025; pp 75–103

[157] Rosca C.-M., Stancu A. (2025). Integration of AI in
Self-Powered
IoT Sensor Systems. Appl. Sci..

[158] Qiu H.-J., Song W.-Z., Wang X.-X., Zhang J., Fan Z., Yu M., Ramakrishna S., Long Y.-Z. (2019). A calibration-free
self-powered sensor for vital sign monitoring and finger tap communication
based on wearable triboelectric nanogenerator. Nano Energy.

[159] Pu X., Liu M., Chen X., Sun J., Du C., Zhang Y., Zhai J., Hu W., Wang Z. L. (2017). Ultrastretchable,
transparent triboelectric nanogenerator as electronic skin for biomechanical
energy harvesting and tactile sensing. Sci.
Adv..

[160] Yu Y., Li Z., Wang Y., Gong S., Wang X. (2015). Sequential
infiltration synthesis of doped polymer films with tunable electrical
properties for efficient triboelectric nanogenerator development. Adv. Mater..

[161] Cui N., Dai C., Liu J., Gu L., Ge R., Du T., Wang Z., Qin Y. (2020). Increasing
the output charge quantity
of triboelectric nanogenerators via frequency multiplication with
a multigap-structured friction layer. Energy
Environ. Sci..

[162] Kong X. Y., Ding Y., Yang R., Wang Z. L. (2004). Single-crystal
nanorings formed by epitaxial self-coiling of polar nanobelts. Science.

[163] Zhao K., Wang Z. L., Yang Y. (2016). Self-powered
wireless
smart sensor node enabled by an ultrastable, highly efficient, and
superhydrophobic-surface-based triboelectric nanogenerator. ACS Nano.

[164] Li C., Guo C., Fitzpatrick V., Ibrahim A., Zwierstra M. J., Hanna P., Lechtig A., Nazarian A., Lin S. J., Kaplan D. L. (2020). Design of biodegradable,
implantable devices towards
clinical translation. Nat. Rev. Mater..

[165] Lu L., Ding W., Liu J., Yang B. (2020). Flexible PVDF-based
piezoelectric nanogenerators. Nano Energy.

[166] Arab Hassani F., Shi Q., Wen F., He T., Haroun A., Yang Y., Feng Y., Lee C. (2020). Smart Materials
for Smart Healthcare—Moving from Sensors and Actuators to Self-Sustained
Nanoenergy Nanosystems. Smart Mater. Med..

[167] Guo R., Fang Y., Wang Z., Libanori A., Xiao X., Wan D., Cui X., Sang S., Zhang W., Zhang H., Chen J. (2022). Deep learning
assisted body area triboelectric hydrogel sensor network
for infant care. Adv. Funct. Mater..

[168] Shi B., Liu Z., Zheng Q., Meng J., Ouyang H., Zou Y., Jiang D., Qu X., Yu M., Zhao L. (2019). Body-integrated self-powered system for wearable
and implantable
applications. ACS Nano.

[169] Zheng Q., Shi B., Li Z., Wang Z. L. (2017). Recent
progress on piezoelectric and triboelectric energy harvesters in biomedical
systems. Adv. Sci..

[170] Azimi S., Golabchi A., Nekookar A., Rabbani S., Amiri M. H., Asadi K., Abolhasani M. M. (2021). Self-powered
cardiac pacemaker by piezoelectric polymer nanogenerator implant. Nano Energy.

[171] Zhao D., Zhuo J., Chen Z., Wu J., Ma R., Zhang X., Zhang Y., Wang X., Wei X., Liu L. (2021). Eco-friendly in-situ gap generation of no-spacer
triboelectric
nanogenerator for monitoring cardiovascular activities. Nano Energy.

[172] Bonnevialle, P. ; Clément, D. Clinical Applications of Bone Substitutes. In Biomechanics and Biomaterials in Orthopedics; Poitout, D. G. , Ed.; Springer: London, 2004

[173] Dai K., Deng S., Yu Y., Zhu F., Wang J., Liu C. (2022). Construction of developmentally inspired
periosteum-like tissue for
bone regeneration. Bone Res..

[174] Ali M., Bathaei M. J., Istif E., Karimi S. N. H., Beker L. (2023). Biodegradable
piezoelectric polymers: recent advancements in materials and applications. Adv. Healthc. Mater..

[175] Kim D., Lee B., Marshall B. P., Jang E., Thomopoulos S., Jun Y. S. (2020). Pulsed electrical stimulation enhances body fluid transport
for collagen biomineralization. ACS Appl. Bio
Mater..

[176] Wang L., Chen K., Fan Y., Yin L. (2022). Novel implantable
devices delivering electrical cues for tissue regeneration and functional
restoration. Med. Nov. Technol. Devices.

[177] Kaynak D., Meffert R., Günhan M., Günhan O. ¨. (2005). A histopathologic investigation on
the effects of electrical stimulation on periodontal tissue regeneration
in experimental bony defects in dogs. J. Periodontol..

[178] Ahn A. C., Grodzinsky A. J. (2009). Relevance
of collagen piezoelectricity
to “Wolff’s Law”: a critical review. Med. Eng. Phys..

[179] Chen L., Chen Z., Zhang M. (2001). Piezoelectric
ceramic--a
novel material for bone replacement. Med. Eng.
Phys..

[180] Heng B. C., Bai Y., Li X., Lim L. W., Li W., Ge Z., Zhang X., Deng X. (2023). Electroactive biomaterials
for facilitating bone defect repair under pathological conditions. Adv. Sci..

[181] Wang A., Liu Z., Hu M., Wang C., Zhang X., Shi B., Fan Y., Cui Y., Li Z., Ren K. (2018). Piezoelectric nanofibrous scaffolds
as in vivo energy
harvesters for modifying fibroblast alignment and proliferation in
wound healing. Nano Energy.

[182] Shuai C., Wang Z., Yang F., Zhang H., Liu J., Feng P. (2024). Laser additive manufacturing of shape memory biopolymer
bone scaffold: 3D conductive network construction and electrically
driven mechanism. J. Adv. Res..

[183] Zhang Y., Xu L., Liu Z., Cui X., Xiang Z., Bai J., Jiang D., Xue J., Wang C., Lin Y., Li Z. (2021). Self-Powered Pulsed
Direct Current Stimulation System for Enhancing Osteogenesis in MC3T3-E1. Nano Energy.

[184] Dong X., Liu F., Wang L., Xu L., Pan H., Qi J. (2023). Nanogenerators for biomedical applications. Mater. Today Comm..

[185] Strangis G., Labardi M., Gallone G. (2024). 3D Printed
Piezoelectric
BaTiO_3_/Polyhydroxybutyrate Nanocomposite Scaffolds for
Bone Tissue Engineering.. Bioengineering.

[186] Kang D., Liu Z., Qian C., Huang J., Zhou Y., Mao X., Qu Q., Liu B., Wang J., Hu Z., Miao Y. (2023). 3D bioprinting of a
gelatin-alginate hydrogel for tissue-engineered hair follicle regeneration. Acta Biomater..

[187] Yao G., Jiang D., Li J., Kang L., Chen S., Long Y., Wang Y., Huang P., Lin Y., Cai W. (2019). Self-activated electrical stimulation for effective
hair regeneration via a wearable omnidirectional pulse generator. ACS Nano.

[188] Zhao D., Zhang K., Meng Y., Li Z., Pi Y., Shi Y., You J., Wang R., Dai Z., Zhou B. (2022). Untethered triboelectric patch for wearable smart sensing
and energy harvesting. Nano Energy.

[189] Wang C., He G., Zhao H., Lu Y., Jiang P., Li W. (2024). Enhancing deep-seated melanoma therapy
through wearable self-powered microneedle patch. Adv. Mater..

[190] Du S., Zhou N., Xie G., Chen Y., Suo H., Xu J., Tao J., Zhang L., Zhu J. (2021). Surface-engineered
triboelectric nanogenerator patches with drug loading and electrical
stimulation capabilities: Toward promoting infected wounds healing. Nano Energy.

[191] Oh J. Y., Bao Z. (2019). Second skin enabled
by advanced electronics. Adv. Sci..

[192] Chortos A., Bao Z. (2014). Skin-inspired electronic devices. Mater. Today.

[193] Liu D., Zhu P., Zhang F., Li P., Huang W., Li C., Han N., Mu S., Zhou H., Mao Y. (2023). Intrinsically
stretchable polymer semiconductor based electronic skin for multiple
perceptions of force, temperature, and visible light. Nano Res..

[194] Liu T., Liu M., Dou S., Sun J., Cong Z., Jiang C., Du C., Pu X., Hu W., Wang Z. L. (2018). Triboelectric-nanogenerator-based soft energy-harvesting
skin enabled by toughly bonded elastomer/hydrogel hybrids. ACS Nano.

[195] Li T., Wei H., Zhang Y., Wan T., Cui D., Zhao S., Zhang T., Ji Y., Algadi H., Guo Z. (2023). Sodium alginate reinforced polyacrylamide/xanthan gum
double network ionic hydrogels for stress sensing and self-powered
wearable device applications. Carbohydr. Polym..

[196] Rana S. M. S., Zahed M. A., Rahman M. T., Salauddin M., Lee S. H., Park C., Maharjan P., Bhatta T., Shrestha K., Park J. Y. (2021). Cobalt-Nanoporous Carbon Functionalized
Nanocomposite-Based Triboelectric Nanogenerator for Contactless and
Sustainable Self-Powered Sensor. Adv. Funct.
Mater..

[197] Zhou K., Zhao Y., Sun X., Yuan Z., Zheng G., Dai K., Mi L., Pan C., Liu C., Shen C. (2020). Ultra-stretchable triboelectric nanogenerator
as high-sensitive
and self-powered electronic skins for energy harvesting and tactile
sensing. Nano Energy.

[198] Périard J. D., Eijsvogels T. M., Daanen H. A. (2021). Exercise under heat
stress: thermoregulation, hydration, performance implications, and
mitigation strategies. Physiol. Rev..

[199] Li W., Lu L., Kottapalli A. G. P., Pei Y. (2022). Bioinspired sweat-resistant
wearable triboelectric nanogenerator for movement monitoring during
exercise. Nano Energy.

[200] Zhi C., Wu H., Hu J. (2024). In-situ welding and thermal activation
enabled robust nanofibers based triboelectric nanogenerator for sustainable
energy harvesting. Nano Energy.

[201] Amieva H., Ouvrard C., Giulioli C., Meillon C., Rullier L., Dartigues J. F. (2015). Self-reported hearing loss, hearing
aids, and cognitive decline in elderly adults: a 25-year study. J. Amer. Geri. Soc..

[202] Guo H., Pu X., Chen J., Meng Y., Yeh M. H., Liu G., Tang Q., Chen B., Liu D., Qi S. (2018). A highly sensitive, self-powered triboelectric auditory sensor for
social robotics and hearing aids. Sci. Robot..

[203] Zheng J., Yu Z., Wang Y., Fu Y., Chen D., Zhou H. (2021). Acoustic core–shell resonance
harvester for application of artificial cochlea based on the piezo-triboelectric
effect. ACS Nano.

[204] Zheng Q., Shi B., Fan F., Wang X., Yan L., Yuan W., Wang S., Liu H., Li Z., Wang Z. L. (2014). In vivo powering of pacemaker by breathing-driven implanted
triboelectric nanogenerator. Adv. Mater.:Compos.
Carbon, Pap. Symp..

[205] Sheng F., Zhang B., Zhang Y., Li Y., Cheng R., Wei C., Ning C., Dong K., Wang Z. L. (2022). Ultrastretchable organogel/silicone fiber-helical sensors
for self-powered implantable ligament strain monitoring. ACS Nano.

[206] Arab Hassani F., Mogan R. P., Gammad G. G., Wang H., Yen S. C., Thakor N. V., Lee C. (2018). Toward self-control
systems for neurogenic underactive bladder: a triboelectric nanogenerator
sensor integrated with a bistable micro-actuator. ACS Nano.

[207] Li J., Long Y., Yang F., Wang X. (2020). Respiration-driven
triboelectric nanogenerators for biomedical applications. EcoMat.

[208] Amrutha B., Prasad G., Sathiyanathan P., Reza M. S., Kim H., Pathak M., Prabu A. A. (2023). Fabrication
of CuO-NP-doped PVDF composites based Electrospun triboelectric nanogenerators
for wearable and biomedical applications. Polymers.

[209] Huang Y. J., Chung C. K. (2023). Design and fabrication of polymer
triboelectric nanogenerators for self-powered insole applications. Polymers.

